# Effects of Naringenin in Preclinical Models of Breast Cancer

**DOI:** 10.3390/biom16030480

**Published:** 2026-03-23

**Authors:** Emily C. Irwin, Newman Siu Kwan Sze, Evangelia Tsiani

**Affiliations:** 1 Department of Health Sciences, Faculty of Applied Health Sciences, Brock University, St. Catharines, ON L2S 3A1, Canada; ei19oy@brocku.ca (E.C.I.); nsze@brocku.ca (N.S.K.S.); 2 Centre for Bone and Muscle Health, Brock University, St. Catharines, ON L2S 3A1, Canada

**Keywords:** breast cancer, naringenin, apoptosis, proliferation, metastasis

## Abstract

Breast cancer is the most commonly diagnosed cancer among women, with approximately one in eight women developing the disease during their lifetime. Despite advancements in current treatment options, breast cancer was responsible for an estimated 670,000 deaths worldwide in 2022. This highlights the urgent need for the development of novel therapeutic strategies. Historically, plant-derived compounds have played a significant role in cancer therapy, exemplified by widely used chemotherapeutic agents such as paclitaxel and docetaxel. In recent years, increasing attention has been directed toward novel plant-derived compounds as potential anti-cancer agents. Among these, Naringenin, a flavonoid predominantly found in citrus fruits, has shown promising antioxidant, anti-inflammatory, and anti-cancer properties. This review highlights recent studies investigating the effects of Naringenin and its derivatives on breast cancer. Evidence from both in vitro and in vivo animal models suggests that Naringenin may exert anti-tumor activity by inhibiting cell proliferation, promoting apoptosis, modulating key cell signaling pathways, and enhancing radio-sensitivity in breast cancer cells. Although preclinical evidence strongly supports the anticancer potential of Naringenin in breast cancer, comprehensive clinical studies are urgently needed to validate its efficacy and safety in humans.

## 1. Introduction

Breast cancer is the second most commonly diagnosed cancer and the second leading cause of cancer-related death among women worldwide, with approximately one in eight women diagnosed during their lifetime [1]. Despite advances in early detection and treatment, breast cancer accounted for an estimated 670,000 deaths globally in 2022 and, in 2025, was the second leading cause of cancer death among women living in Canada, underscoring the continued need for improved therapeutic strategies [1,2]. The disease is highly heterogeneous and is classified into four major molecular subtypes based on the expression of estrogen receptor (ER), progesterone receptor (PR), and human epidermal growth factor receptor 2 (HER2), and differs in prognosis, clinical behavior, and therapeutic responsiveness, necessitating individualized treatment approaches [3]. Luminal A (ER+/PR+/HER2−) is the most common subtype, accounting for approximately 40–50% of new cases, and is typically characterized by low proliferation and the most favorable prognosis; treatment primarily relies on endocrine therapy, sometimes with limited chemotherapy depending on risk [4,5]. Luminal B (ER+/PR+/HER2±) represents 20–30% of cases and has higher proliferation and a worse prognosis than Luminal A; treatment often involves endocrine therapy combined with chemotherapy, and HER2-targeted therapy if HER2 is overexpressed [5]. HER2-enriched breast cancer (ER−/PR−/HER2+) accounts for roughly 10–15% of cases and is driven by amplification of the HER2 signaling pathway; it is treated with HER2-targeted agents combined with chemotherapy [5]. Triple-negative breast cancer (ER−/PR−/HER2−) comprises approximately 10–15% of breast cancers and is generally more aggressive with fewer targeted treatment options; management typically involves systemic chemotherapy, although immunotherapy and PARP inhibitors have expanded treatment options for select patients [5].

Key hallmarks of cancer, including sustained proliferation, evasion of apoptosis, and enhanced metastatic potential, are often driven by dysregulation of signaling pathways that control cell survival and homeostasis [6]. The most common mutations and signaling defects in breast cancer involve alterations in pathways that drive uncontrolled cell growth, survival, and genomic instability [6]. Well-known hereditary mutations, most prominently BRCA1 and BRCA2, which are widely recognized even by the general public, impair homologous recombination-mediated DNA repair, predisposing individuals to genomic instability and increased breast cancer risk [6]. Beyond BRCA-associated disease, somatic alterations frequently affect key oncogenic signaling networks [6]. PIK3CA mutations activate the PI3K–AKT–mTOR pathway [7,8], enhancing proliferation and therapy resistance, and TP53 loss, which disrupts genome surveillance, DNA repair, and cell cycle control [7,9]. Amplification or overexpression of HER2 drives excessive receptor tyrosine kinase signaling, and aberrant activation of other growth factor receptors such as epidermal growth factor receptor (EGFR)/human epidermal growth factor receptor (HER1) further stimulates downstream phosphatidylinositol 3-kinase (PI3K)/AKT and Ras/mitogen-activated protein kinase (MAPK) cascades that promote tumor growth, survival, and therapeutic resistance [6,7,10]. Additional contributors include dysregulation of hormone receptor signaling (estrogen and progesterone receptors), alterations in the MAPK pathway, and defects in cell cycle regulators [6,11]. Impairments in intrinsic and extrinsic apoptotic pathways, involving caspases and BCL-2 family proteins [12], also support cancer cell survival and resistance to treatment [7,13]. Collectively, these genetic and signaling alterations contribute to the initiation and progression of breast cancer and substantially influence the varied responses to therapeutic interventions.

Current breast cancer therapies encompass a range of options, including surgery, chemotherapy, radiation, hormone therapy, and targeted therapy [7]. While these treatments have significantly improved survival rates, their efficacy is often limited by intrinsic or acquired resistance, as well as toxicity [7]. Thus, there is an urgent need for novel therapeutics that can enhance or complement existing treatments while minimizing adverse effects.

In recent years, natural compounds derived from plants have gained increasing attention for their potential as anti-cancer agents. Several chemotherapeutic drugs currently in clinical use are plant-derived and have shown substantial efficacy in breast cancer treatment [14,15]. Among emerging phytochemicals, flavonoids, a class of polyphenolic compounds found in fruits and vegetables, have demonstrated promising anti-cancer activities [16]. One such compound, Naringenin, is a naturally occurring flavonoid (C_15_H_12_O_5_, MW 272.26 g/mol) found abundantly in citrus fruits such as oranges and grapefruits [17]. Naringenin (Nar) possesses antioxidant, anti-inflammatory, and anti-cancer properties, and has been shown to modulate various signaling pathways associated with cancer cell proliferation, apoptosis, and metastasis [17]. Although current published reviews on Nar and breast cancer [18] have summarized the use of Nar in breast cancer in broad terms, our review provides a more in-depth analysis of individual studies, critically evaluating mechanistic findings and experimental limitations to better assess the translational potential of Nar.

This review aims to explore the current evidence on the potential of Naringenin as a therapeutic agent in breast cancer, with a particular focus on its molecular mechanisms of action and its role in modulating key signaling pathways involved in tumor development and progression.

We gathered evidence from PubMed and Google Scholar using key words: “Naringenin and Breast Cancer” and reviewed in vitro cell culture models and in vivo animal models. The majority of studies are peer reviewed, and articles excluded were not specific to the topic, not available in English, or published earlier than 2010. All studies meeting the inclusion criteria are presented in chronological order and include breast cancer treatment with Naringenin alone, Naringenin analogs, and nanoparticles, and in combination with current treatments (chemotherapy, radiotherapy, hormone therapy, and plant-derived antioxidants).

## 2. Naringenin Against Breast Cancer: In Vitro Studies

Studies that have examined the effect of Nar in cultured breast cancer cells are presented below and in Table 1.

Nar treatment of MCF-7 luminal A breast cancer cells resulted in decreased aromatase or cytochrome P450 (CYP19) activity, the enzyme catalyzing the rate-limiting step in estrogen synthesis [19] (Table 1). Similarly, Nar treatment decreased CYP19 activity of recombinant human CYP19 supersomes, microsomes from the endoplasmic reticulum of baculovirus-transfected insect cells with high levels of human CYP19 [19]. RT-qPCR analysis revealed no change in CYP19 mRNA levels in MCF-7 cells treated with Nar [19]. These results indicate the potential of Nar to inhibit CYP19 activity in MCF-7 breast cancer cells without affecting CYP19 protein levels. Unfortunately, this inhibitory effect of CYP19 was not associated with a functional assay to indicate decreased cell viability or proliferation. Further research is needed to investigate the mechanisms behind these effects.

In MCF-7 and T47D luminal A breast cancer cells, expressing only ERα, Nar treatment decreased cell proliferation, whereas MDA-MB-231 triple-negative breast cancer (TNBC) cells had no change in cell proliferation [20]. Additionally, in MCF-7 and T47D cells treated with Nar and an ER inhibitor, the previous decrease in cell proliferation was reduced, indicating Nar partially acts through ERα to decrease cell proliferation [20]. Bisphenol A (BPA) is an established inducer of cancer growth through ERα activation, and a competitive radiometric binding assay revealed Nar has a higher affinity for the ERα receptor than BPA, indicating the potential of Nar to inhibit BPA-induced activation of ERα even in high BPA concentrations [20]. Additionally, Nar treatment of MCF-7 and T47D breast cancer cells in combination with BPA resulted in decreased cell proliferation, Akt phosphorylation, and Bcl-2 protein levels, and increased activation of caspase 3 and cleaved PARP protein levels, indicating increased apoptosis. Furthermore, in MCF-7 and T47D cells treated with Nar, immunoblotting revealed increased phosphorylated p38, a protein phosphorylated in the caspase 3 apoptotic cascade; however, in cells treated with a p38 inhibitor, SB 203580, the antiproliferative effects of Nar were abolished [20]. Overall, these data indicate the potential of Nar to inhibit BPA induced ERα activation and cell proliferation in ERα-positive breast cancer cells.

MDA-MB-231 and MDA-MB-468 TNBC cells treated with Nar resulted in decreased cell viability [21].

Exposure of MDA-MB-468 and MDA-MB-231 breast cancer cells to Nar (extracted from *Thymus vulgaris*) resulted in a dose and time-dependent decrease in cell viability and colony formation, whereas Nar did not have a significant effect on CRL1554 normal human fibroblast cells [22]. Nar increased the proportion of cells in S-phase and G2/M phase while decreasing the proportion of cells in G0/G1-phase of the cell cycle in both breast cancer cell lines. Flow cytometry revealed an increase in early apoptosis, late apoptosis, and necrosis of breast cancer cells treated with Nar. Western blot analysis revealed decreased phosphorylation of IκB, an upstream mediator of NF-κB function, and decreased levels of NF-κB p65 protein, indicating decreased NF-κB activation and nuclear translocation. Additionally, Nar treatment decreased phosphorylated Akt levels in breast cancer cell lines. qRT-PCR analysis revealed a significant increase in tumor suppressor (p18, p19, and p21) mRNA levels and decreased cell cycle progression (cyclin-dependent kinase (Cdk) 4, Cdk6, and Cdk7) mRNA levels with Nar treatment. Additionally, Nar treatment increased pro-apoptotic mRNA levels (Bak, caspases-3, 7, 8, 9, apoptosis-inducing factor (AIF), and Bax), and decreased anti-apoptotic mRNA levels (Bcl2, xIAP, and c-IAP-2). Overall, these data indicate a strong anticancer potential of Nar in breast cancer cells while sparing normal human fibroblast cells.

In silico analysis indicated Nar has a high binding energy for the ATP binding site of the tyrosine kinase (TK) domain of human epidermal growth factor receptor 2 (HER2), and the binding energy was comparable to established HER-TK inhibitors SYR and lapatinib [23]. A kinase activity assay confirmed the in silico evidence demonstrating Nar dose dependently decreases HER2 activity [23]. Furthermore, in HER2-overexpressing SKBr3 breast cancer cells, Nar treatment resulted in decreased cell viability and mitochondrial membrane potential, a sign of early apoptosis, and increased chromatin condensation, a sign of late apoptosis [23]. Unexpectedly, Nar exhibited similar effects in the HER2-negative MDA-MB-231 and MCF-7 breast cancer cell lines, although apoptotic events occurred at earlier time points in the HER2-positive SKBr3 cells. Western blotting analysis revealed increased cleaved caspase 8 protein levels and decreased pro-caspase 3 protein levels, further confirming Nar-induced apoptosis in SKBr3 cells [23]. However, the active, cleaved form of caspase 3 was not analyzed. Additionally, Nar treatment of SKBr3 cells resulted in increased S phase cell cycle arrest [23]. This study provides evidence of the strong potential of Nar to target and increase apoptosis of HER2-positive breast cancer cells. Further research is needed to investigate other potential anti-proliferative mechanisms of Nar in HER2-negative breast cancer cells.

Treatment of the tamoxifen-resistant human MCF-7/TAMR-1 breast cancer cell line with Nar decreased cell proliferation [24]. Treatment with Nar or U0126, a MEK inhibitor, resulted in decreased cell density and viability after 96 h, with combination treatment resulting in a greater effect [24]. Additionally, flow cytometry analysis revealed that when Nar and U0126 treatments are combined, decreased concentrations of Nar and Tam are required to impair cell proliferation and viability [24]. Although all treatments increased apoptosis to a similar degree, Nar treatment decreased pro-caspase-7, pro-caspase-9, and cleaved poly (ADP-ribose) polymerase (PARP) with greater reduction when treated in combination with U0126; these results suggest an induction of apoptosis; however, the authors did not measure levels of the cleaved, active form of caspase-7 or 9 [24]. Despite no change in overall expression of estrogen receptor alpha (ERα), immunofluorescence revealed Nar treatment alone and in combination resulted in increased ERα peri-nuclear localization, whereas no change was observed in U0126-treated cells [24]. These findings suggest Nar induces apoptosis through caspase-7, caspase-9, and PARP activation, and alters the localization of ERα.

Transforming growth factor beta (TGF-β1) increased breast cancer metastasis, and previous data in vivo indicate that Nar treatment decreases TGF-β1 secretion from breast cancer tumors and decreases metastasis. These data were confirmed in vitro, where Nar treatment of 4T1-Luc2 breast cancer cells transfected to overexpress TGF-β1 (4T1-TGF-β1) decreased TGF-β1 secretion and increased intracellular TGF-β1 accumulation [25]. Nar treatment did not affect transcription, post-transcriptional regulation, translation, or lysosomal degradation of TGF-β1, further indicating intracellular accumulation with no effect on TGF-β1 synthesis or degradation [25]. To investigate the intracellular accumulation, it was hypothesized that the transport of TGF-β1 may be affected by Nar [25]. TGF-β proteins are synthesizes a precursor proteins and cleaved in the endoplasmic reticulum, then transported through the trans Golgi network (TGN) to the plasma membrane [25]. Protein kinase C (PKC) is involved in the transport and sorting of many proteins from the TGN to the cell membrane [25]. Increased Tgf-β1 levels increased PRKCE and PRKCZ mRNA levels, indicating that PKC-ε and PKC-ζ may be involved in TGF-β1 transportation [25]. Nar treatment decreased phosphorylation of PKC-ε and PKC-ζ, with no change in mRNA and protein levels [25]. siRNA PKC-ε and PKC-ζ knockdown in 4T1 breast cancer cells had similar results to Nar treatment, and when treated in combination with Nar, there was a synergistic effect, resulting in a greater increase in intracellular and a decrease in secreted TGF-β1 [25]. Additionally, phorbol 12-myristate 13-acetate (PMA) treatment, a PKC activator, in 4T1 breast cancer cells increased cytoplasmic distribution of TNG and decreased intracellular concentrations of TGF-β1 and increased concentrations of TGF-β1 in the media, when Nar was used in combination these affects were partially attenuated [25]. Furthermore, treatment with calphostin C, a specific PKC inhibitor, increased TGN and TGF-β1-containing vesicles near the nucleus, increased intracellular TGF-β1 accumulation, and decreased TGF-β1 levels in the media, further implicating PKC in TGF-β1 transport [25]. Overall, these data indicate that Nar reduces TGF-β1 trafficking and secretion through decreasing PKC phosphorylation.

E0771 luminal B breast cancer cells treated with Nar decreased cell viability and increased sub-G1 cycle arrest, indicating increased apoptosis [26]. Nar treatment increased Thr172 phosphorylation of AMPK and decreased cyclin D1 protein levels, with no difference in Bax and Bcl-2 protein levels [26]. Overall, these data indicate the potential of Nar to inhibit E0771 breast cancer cell viability and growth in vitro.

ERα antagonists are attractive targets in estrogen-positive breast cancer, as activation of ERα leads to increased proliferation of breast cancer cells [27]. Ligand-based machine learning indicated that Nar has a strong potential to target ERα. This was further indicated by molecular docking, which revealed a weak binding affinity of Nar for ERα [27]. A luciferase reporter gene assay was used to confirm the potential of Nar to bind to ERα and revealed weak antiestrogen effects and weak decreased ERα activity [27]. These data indicate the weak potential of Nar to decrease ERα activity, indicating potential anti-breast cancer effect; however, these results need further investigation to confirm the antagonistic potential of Nar.

Nar treatment decreased the viability of MDA-MB-231 and MCF-10A TNBC cells. Flow cytometry analysis revealed Nar treatment increased cell cycle arrest at the G0/G1 phase and increased apoptosis of MDA-MB-231 cells [28]. Apoptosis was further indicated in MDA-MB-231 cells by increased caspase-3/7 activity, increased fragmented DNA, and reduced NF-kB DNA binding [28]. These results indicate that Nar exerts its anti-breast cancer effects by increasing apoptosis and cell cycle arrest.

In MCF-7 and T47D ER+ breast cancer cells, Nar treatment significantly increased death-associated factor 6 (DAXX) protein levels; DAXX plays an important role in inhibiting tumor-initiating cells (TICs) [29]. Additionally, Nar treatment prevented cell proliferation and, importantly, did not increase TFF1 (PS2) transcripts, indicating that there was no increase in classical ER signaling [29]. These results indicate that Nar treatment can prevent ER+ breast cancer cell proliferation and prevent ER-stimulated cell growth and survival, while activating cascades involved in tumor suppression. Furthermore, incubation of MCF-7 and T47D with fulvestrant, an ER inhibitor, prevented the Nar-induced increase in DAXX protein levels, indicating that the ER is required for DAXX protein expression [29]. Additionally, in MCF-7 cells, the half-life of DAXX is increased by Nar treatment, and fulvestrant treatment abolishes this effect, further indicating that this effect of Nar is dependent on ER [29]. Nar treatment of MCF-7 and T47D cells inhibited mammosphere formation, indicating decreased TIC, whereas in MCF-7 and T47D cells incubated with fulvestrant, the Nar-induced inhibition of mammosphere formation was prevented [29]. Additionally, in MCF-7 and T47D cells with siRNA-mediated knockdown of DAXX, the Nar-induced inhibition of mammosphere formation was again prevented [29]. These data further indicate the role of the ER and DAXX in Nar-induced prevention of mammosphere formation. Additionally, in cells incubated with a selective ERα agonist, propyl pyrazole triol (PPT), or an ERβ agonist, diarylpropionitrile (DPN), the increase in DAXX protein levels was comparable; however, the increase in DAXX protein was less than the Nar-induced increase in DAXX protein levels, indicating the activation of both ERα and ERβ isoforms may be required for Nar-induced increase in DAXX protein levels [29]. Additionally, in both MCF-7 and T47D cells, PHTPP, a selective ERβ antagonist, prevented Nar-induced increase in DAXX protein levels and inhibition of mammosphere formation. Whereas in cells treated with MPP, a selective ERα antagonist, the Nar-induced increase in DAXX protein levels and inhibition of mammosphere formation was completely abolished in MCF-7 cells and only partially attenuated in T47D cells [29]. DAXX potently inhibits TICs and NOTCH signaling; therefore, the effects of Nar on NOTCH4 and NOTCH gene targets were investigated. Nar treatment increased DAXX protein levels while NOTCH4 protein levels and mRNA of HES1 and HEY1, downstream gene targets of NOTCH, were undetectable [29]. However, in siRNA DAXX knockdown cells, Nar was not able to prevent NOTCH4 protein level or mRNA HES1, and HEY1 increases [29]. Additionally, in MCF-7 and T47D breast cancer cells, Nar decreased SOX2, OCT4, and NANOG mRNA proteins associated with TICs; these effects were completely abolished in DAXX knockout cells. Overall, these results indicate that Nar may be an attractive therapeutic agent to prevent ER+ breast cancer cell growth and mammosphere formation through the activation of DAXX and inhibition of NOTCH signaling, and may prevent ER-mediated cell growth and survival.

In MCF-7 breast cancer cells, Nar treatment inhibited mammosphere formation and decreased cell viability [30]. Additionally, Nar treatment decreased colony formation to a similar degree as doxorubicin (DOX), an established chemotherapeutic, and decreased cell migration. In both MCF-7 cells and breast cancer stem cell (BCSC) mammospheres, Nar treatment resulted in G1 cell cycle arrest and increased early and late-stage apoptosis [30]. Furthermore, Nar treatment reduced mRNA levels of proteins associated with breast cancer cell proliferation, invasion, and metastases, such as ß-catenin, MMP9, and ALDH1, while increasing mRNA levels of Bax, a pro-apoptotic protein [30]. Similarly, in mammospheres, Nar treatment reduced mRNA levels of Vimentin, a protein associated with breast cancer metastases, ALDH1, and Bcl2 [30]. In both MCF-7 and mammospheres, Nar treatment increased tumor suppressor p53 and, surprisingly, estrogen receptor 1 (ESR1) mRNA levels [30]. These results indicate the anti-breast cancer potential of Nar to decrease cell viability, migration, and increase apoptosis.

The Valencia orange, *C. aurantifolia*, yielded Nar as one of its main components and significantly decreased cell viability in MCF-7 and T47D breast cancer cells, requiring a significantly higher concentration to decrease cell viability of normal human HFB4 cells [31]. Furthermore, Nar treatment alone required lower concentrations to decrease cell viability in breast cancer cells and did not significantly decrease the cell viability of normal human HFB4 cells [31]. Unfortunately, the length of exposure of the cells to Nar is not clear. Molecular docking of Nar with aromatase enzyme suggests favorable binding with the active site, indicating potential inhibitory effects of Nar against aromatase enzyme [31]. Additionally, molecular docking revealed favorable docking of Nar with the ER and may act as a modulator of ER activity [31]. Overall, although this evidence suggests the potential of Nar to decrease ER+ breast cancer cell viability by decreasing aromatase activity, further investigation is required to confirm whether this is true in vivo.

Nar treatment of MDA-MB-231 breast cancer cells significantly decreased cell viability, colony formation, and cell migration and invasion [32]. Visual apoptosis analysis by AO/EB staining revealed increased damaged DNA, membrane blebbing, nucleus condensation, and membrane rupture, indicating increased apoptosis [32]. To further investigate apoptosis, Annexin V/PI and flow cytometry were used and confirmed increased apoptosis in MDA-MB-231 cells treated with Nar [32]. Proteins involved in apoptosis were assessed using Western blotting, which revealed increased caspase-3, -8, -9, and BAX protein levels and decreased Bcl-2 protein levels, further indicating increased apoptosis; however, the active form of these caspases was not identified [32]. Additionally, cell cycle analysis revealed increased G2/M phase arrest, indicating Nar treatment can halt the cell cycle at the G2/M phase in MDA-MB-231 breast cancer cells [32]. Overall, these results indicate that Nar treatment can decrease cell viability, colony formation, and cell migration and can increase apoptosis of MDA-MB-231 breast cancer cells, making it a potential novel anti-cancer therapeutic; however, further in vivo studies are required to confirm these results.

Analysis using the TISIDB database and TIMER database found that upregulation of FKBP4 protein and downregulation of NRF2 are associated with luminal A and basal-like subtype of breast cancers and decreased survival [33]. This association was confirmed in five breast cancer cell lines (MCF7, T47D, BT549, MDA231, and SKBr3); however, this association was most prominent in T47D, luminal A subtype, and BT549, basal-like subtype, cell lines. Nar treatment decreased cell proliferation and colony formation in T47D and BT549 breast cancer cell lines [33]. Western blot analysis revealed Nar treatment of T47D and BT549 cell lines decreased FKBP4 protein levels and increased NRF2 protein levels [33]. Knockdown of NRF2 by shRNA in T47D and BT549 attenuated Nar’s decrease in cell proliferation and colony formation, indicating that the effects of Nar on decreased cell proliferation and colony formation are partially due to the FKBP4/NRF2 signaling pathway. Overall, these data indicate the potential of Nar to decrease cell proliferation and colony formation in luminal A subtype and basal-like subtype breast cancer cell lines.

CYP19A1 (aromatase) is an enzyme that catalyzes the conversion of androgens to estrogens and therefore modulating its activity is an attractive target in ER+ breast cancers [34]. In silico analysis revealed Nar and CYP19A1 exhibited a stable conformation, indicating a high likelihood of interaction based on molecular docking analysis, and possessed favorable drug-like physicochemical properties [34]. Additionally, Nar decreased estrogen formation and therefore aromatase activity in an aromatase activity assay. Overall, this study indicates the ability of Nar to inhibit aromatase activity.

The aryl hydrocarbon receptor (AHR) is an intracellular transcription factor; abnormal function of AHR is associated with breast cancer progression, and, therefore, regulation of AHR activity is an attractive target to decrease breast cancer progression. In silico analysis revealed Nar has a high affinity score for the orthosteric site of the AHR [35]. Additionally, in MCF-7 breast cancer cells, canonical activation of AHR induced by tetrachlorodibenzo-p-dioxin (TCDD) treatment increased cytochrome P4501A (CYP1A1) activity; these effects were inhibited by Naringenin in a dose-dependent manner [35]. This study provides evidence indicating the antagonistic potential of Naringenin for the AHR and its potential anti-breast cancer effects.

In silico analysis revealed Nar has a higher binding affinity for the progesterone receptor (PR) compared to progesterone and may be an attractive target to decreased PR induced breast cancer growth and survival [36]. Further research is needed to investigate these potential effects.

Decreasing the activation of estrogen receptor substrate 1 (ERS1) in ER + breast cancers and vascular endothelial growth factor A (VEGFA) is an attractive target to decrease tumor growth and breast cancer proliferation, whereas activation of TP53 can decrease breast cancer development and progression. Molecular docking showed that Naringenin has strong docking capabilities with ERS1, VEGFA, and TP53, and therefore may decrease ERS1 and VEGFA activation and increase TP53 activation [37]. In vitro investigation demonstrated that Naringenin decreased MCF-7 breast cancer proliferation and migration [37]. Additionally, Western blot analysis revealed that Nar treatment significantly decreased ERS1 protein levels [37]. These results indicate the potential of Nar to decrease proliferation, migration, and ERS1 protein levels in ER+ breast cancer cells. Further research is needed to investigate the potential molecular mechanisms behind these effects.

**Figure 1:**
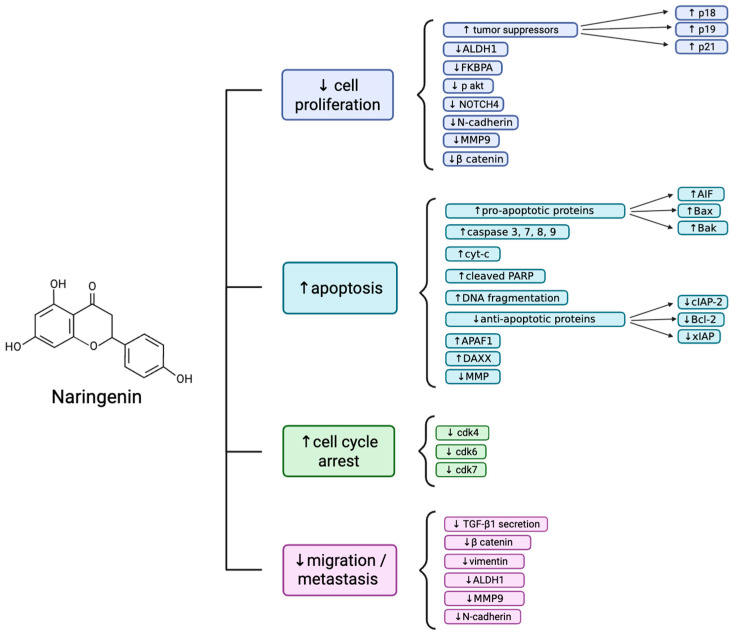
Summary of the anti-breast cancer effects of Naringenin utilizing in vitro models of breast cancer. Created in BioRender. Emily Irwin. (2026) https://BioRender.com/c29m951.

**Figure 2:**
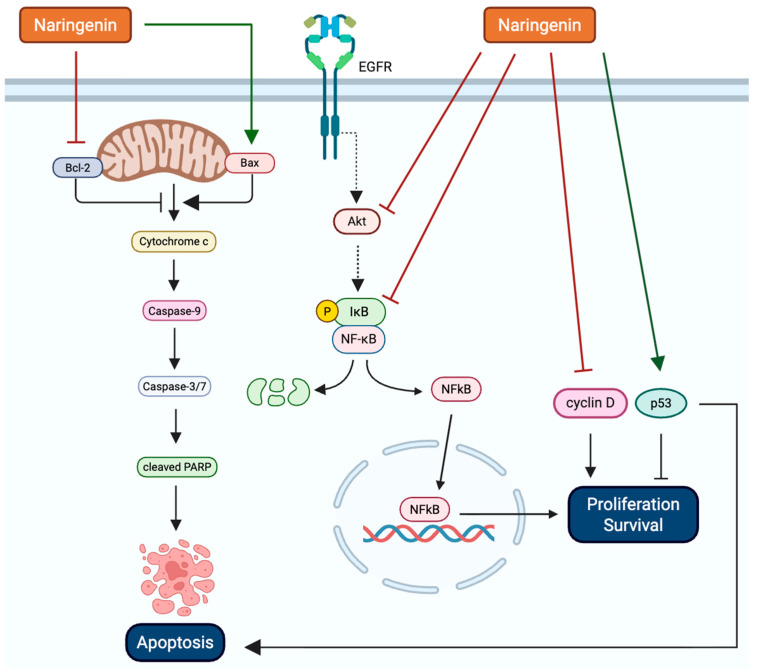
Naringenin decreased proliferation and induced apoptosis of breast cancer cells in vitro. Nar decreased the anti-apoptotic protein Bcl-2 and increased the pro-apoptotic protein Bax, cytochrome C, caspase -3, -7, and -9, and increased cleaved PARP protein levels, leading to apoptosis of breast cancer cells. Furthermore, Nar leads to the activation of p53, inhibition of cell cycle protein cyclin D, inhibition of Akt, and activation of NFkB. Created in BioRender. Emily Irwin. (2026) https://BioRender.com/c29m951.

Overall, Nar induces apoptosis in breast cancer cells through increased pro-apoptotic proteins such as Bak and Bax and decreasing anti-apoptotic proteins including Bcl-2, triggering caspase activation (-3, -7, -8, and -9) and subsequent PARP cleavage [20,22,23,24,28,32] (Figure 1). Nar also increased early and late apoptosis, chromatin condensation, and mitochondrial membrane depolarization in both ERα-positive (MCF-7, T47D) and HER2-positive (SKBr3) cells, as well as TNBC cells (MDA-MB-231, MDA-MB-468), while sparing normal fibroblasts [22,23,28]. Mechanistically, Nar decreases NF-κB and Akt signaling, increases phosphorylated p38 and AMPK, and upregulates apoptosis-related mRNAs (Bak, Bax, caspases, AIF), inducing apoptosis of breast cancer cells [20,22,26,28]. Nar decreases cell viability, proliferation, and survival in ER+, HER2+, and TNBC breast cancer cells while exhibiting minimal toxicity to normal human fibroblasts [21,22,23,26,28,31,33]. These effects are mediated through multiple mechanisms, including downregulation of key cell cycle proteins such as cyclin D1 and Cdk4, Cdk6, and Cdk7, upregulation of tumor suppressors (p18, p19, p21, p53, and DAXX), and inhibition of Akt and NF-κB signaling [22,23,26,28,33]. Consequently, Nar induces cell cycle arrest at G0/G1, S, and G2/M phases [22,23,26,28]. In addition, Nar inhibits breast cancer cell migration and invasion by reducing the expression of metastasis-associated proteins, including Vimentin, MMP9, ALDH1, ERS1, and VEGFA, and by impairing TGF-β1 trafficking through PKC inhibition [25,30,32,37]. Collectively, these studies suggest that Nar exerts anti-cancer effects by targeting multiple pathways, suppressing proliferation, inducing cell cycle arrest, and limiting metastatic potential (Figure 2). Inconsistencies in the role of ER signaling in Nar-mediated effects may be attributed to differences in treatment concentration, exposure duration, and ER isoform expression across breast cancer cell lines. For example, studies using MCF-7 and T47D cells reported ER-dependent effects, potentially due to lower Nar concentrations compared to studies observing ER-independent mechanisms [29]. Similarly, conflicting findings regarding Bax and Bcl-2 protein expression, where some studies report no change [26] while others observe modulation [22,30,32], may reflect differences in breast cancer subtype or cellular context.

**Table 1:** Evidence of the effects of Naringenin against breast cancer: summary of in vitro studies.

Reference	Cell Line	Treatment	Finding	Mechanism
[19]	MCF-7	Nar (0–25 µM) 1–24 h	↓ CYP19 activity	N/A
[20]	MCF-7 and T47D expressing only ERα	Nar alone (1.0 × 10^−9^–1.0 × 10^−4^ M) 1 h or 24 h or in combination with BPA (1.0 × 10^−5^ M) 1 h or 24 h	↓ Cell proliferation	↓ p-Akt protein levels ↓ Bcl-2 protein levels ↑ Activated caspase-3 protein levels ↑Cleaved PARP protein levels ↑ p-p38 protein levels
[21]	MDA-MB-231 and MDA-MB-468	7.81–1000 µg/mL 72 h	↓ Cell proliferation	N/A
[22]	MDA-MB-468 MDA-MB-231	3 mM Nar (extracted from *Thymus vulgaris*), 24–48 h	↓ Cell viability ↓ Proliferation ↓ Colony formation ↑ Apoptosis ↑ Chemosensitivity	↑ S-phase and G2/M phase ↓ G0/G1-phase ↑ Early, late-stage apoptotic, and necrotic cells ↓ p-IκB protein levels ↓ NF-κB p65 protein levels ↓ p-Akt ↑ p18, p19, and p21 mRNA levels ↓ Cdk4, Cdk6, and Cdk7 mRNA levels ↑ Bak, caspases 3, 7, 8, 9, AIF, and Bax mRNA levels ↓ Bcl2, xIAP and c-IAP-2 mRNA levels
[23]	SKBR3	10–250 μM Naringenin for 12–24 h	↓ Cell viability ↑ Apoptosis	↓ Mitochondrial membrane potential ↑ Chromatin condensation ↑ Cleaved caspase 8 ↓ Caspase 3 protein levels ↑ S phase cell cycle arrest
[24]	MCF-7/TAMR-1	200 μM Naringenin 24–96 h or U0126 (10 mM) alone or in combination	↓ Cell viability ↓ Cell density ↑ Apoptosis	↓ Caspase 7 protein ↓ PARP protein ↑ ERα peri-nuclear localization
[25]	4T1-Luc2 transfected to overexpress TGF-β1	Nar 100 μM 24–48 h	↓ TGF-β1 secretion ↑ intracellular TGF-β1	↓ p-PKC-ε and PKC-ζ
[26]	E0771	Nar 50,100, and 200 µM 24, 48, and 72 h	↓ Cell viability	↑ sub-G1 cycle arrest ↑ p-AMPK ↓ Cyclin D1 protein levels
[28]	MDA-MB-231	6.25, 12.5, 25, 50, and 100 µg/mL Nar 12, 24, or 48 h	↓ Cell viability	↑ G0/G1 phase ↑ Caspase-3/7 activity ↑ DNA fragmentation ↓ NF-kB DNA binding
[29]	MCF-7 or T47D	100 nM Nar, 24–72 h	↓ Cell proliferation	↑ DAXX protein levels ↓ Mammosphere formation ↓ NOTCH4 protein levels ↓ HES1 mRNA ↓ HEY1 mRNA
[30]	MCF-7 (2D)	100–200 μM Naringenin (72 h)	↓ Cell viability ↓ Cell migration	↑ G1 phase ↑ Early and late-stage apoptosis ↓ ß-catenin mRNA ↓ MMP9 mRNA ↓ ALDH1 mRNA ↑ Bax mRNA ↑ p53 mRNA ↑ ESR1 mRNA
	MCF-7 (3D) mammospheres	100–200 μM Naringenin (72 h)	↓ Mammosphere formation ↓ Cell migration	↓ Vimentin mRNA ↓ ALDH1 ↓ Bcl2 ↑ p53 mRNA ↑ ESR1 mRNA
[31]	MCF-7 and T47D	0–50 µg/mL Nar	↓ Cell viability	N/A
[32]	MDA-MB-231	Nar 20, 80, and 160 μM, 24 or 48 h	↓ Cell viability ↓ Cell migration ↑ Apoptosis ↓ Colony formation	↑ Damaged DNA ↑ Membrane blebbing ↑ Nucleus condensation ↑ Membrane rupture ↑ Caspase-3, -8, -9 protein levels ↑ BAX protein levels ↓ Bcl-2 protein levels ↑ G2/M phase
[33]	T47D	100 nM Nar 48–72 h	↓ Cell proliferation ↓ Colony formation	↓ FKBP4 protein levels ↑ NRF2 protein levels
	BT549	100 nM Nar 48–72 h	↓ Cell proliferation ↓ Colony formation	↓ FKBP4 protein levels ↑ NRF2 protein levels
[38]	MCF-7	20 or 40 μg/mL SNS	↓ Cell migration ↓ Cell invasion	↓ N-cadherin protein levels ↓ Vimentin protein levels
	E0771	20 or 40 μg/mL SNS	↓ Cell migration ↓ Cell invasion	↓ N-cadherin protein levels ↓ Vimentin protein levels
[35]	MCF-7	Nar 10, 20, or 30 μM for 2 h	↓ CYP1A1 activity	N/A
[37]	MCF-7	100 μM or 150 μM for 48 h	↓ Proliferation ↓ Migration	↓ ERS1 protein levels

Table legend: ↑ Increased, ↓ Decreased, p–phosphorylated.

## 3. Naringenin Analogs and Nanoparticles Against Breast Cancer: In Vitro Studies

Studies that have examined the effect of Nar analogs and nanoparticles in cultured breast cancer cells are presented below and in Table 2.

Saccharomyces cerevisiae yeast, stably transfected with human ERα and a reporter gene, treated with Nar, 8-prenylNaringenin (8PN), or 6-(1,1-dimethylallyl)naringenin (6DMAN), resulted in weak ERα activity [39]. Treatment of the yeast with Nar required significantly higher concentrations to activate ERα than the positive control, 17beta-estradiol (E2); however, treatment with the prenylated Nar (8PN or 6DMAN) required decreased concentrations to activate ERα, indicating prenylation may increase the ability of Nar to activate ERα [39]. MVLN cells, derived from MCF-7 ERα positive breast cancer cells that are stably transfected with a luciferase gene under the control of the estrogen response element, treated with Nar resulted in activation of ERα at high concentrations, whereas treatment with 8PN and 6DMAN resulted in increased ERα activation at much lower concentrations, with 8PN more requiring the lowest concentrations to induce ERα activation and getting similar activation to E2 treated cells [39]. Similar results were indicated with an alkaline phosphatase activity assay in endometrial adenocarcinoma cells. These results indicate that prenylation of Nar increased the ability to activate and bind ERα.

MDA-MB-231 breast cancer cells treated with Copper (II) and 2,2′-bipyridine complexed with Nar (NarCuB) resulted in decreased colony size and formation, proliferation, and migration compared to Nar alone, with limited effects in MCF-10A normal breast cells [40]. Additionally, NarCuB treatment decreased pro-MMP9 protein levels, a marker of cell migration and metastasis, although the active form, MMP9, was not measured [40]. Furthermore, MDA-MB-231 breast cancer cells treated with NarCuB and incubated with DAPI had increased apoptotic nuclei, and flow cytometry revealed that NarCuB treatment increased apoptosis and necrosis [40]. PCR analysis revealed increased caspase-9 mRNA levels with no change in caspase-8 or the anti-apoptotic protein Bcl-2 mRNA levels [40]. These data indicate that NarCuB treatment may increase the effects of Nar on colony size, formation, cell proliferation, and migration; however, interpretation is limited by the use of varying drug concentrations and the lack of consistent responses across treatment concentrations.

A complex of Naringenin and oxidovanadium (IV) with H2O (VOnar) was synthesized, and VOnar treatment of SKBr3 and MDA-MB-231 breast cancer cell lines resulted in a greater decrease in cell viability than Nar alone, with a greater decrease in cell viability in MDA-MB-231 compared to SKBr3 breast cancer cells [41]. Both SKBr3 and MDA-MB-231 treated with Nar alone had no change in intracellular ROS, whereas treatment with VOnar resulted in a 1.5-fold increase [41]. Similarly, VOnar treatment decreased mitochondrial membrane potential (MMP), increased caspase 3/7 activity, H2A histone family member X (H2AX) phosphorylation levels, and lactate dehydrogenase (LDH) levels, indicating increased apoptosis and nuclear damage, with a greater effect in MDA-MB-231 compared to SKBr3 cells, whereas Nar treatment resulted in no change [41]. Furthermore, VOnar treatment but not Nar increased phosphorylated histone H3 (p-H3) in both MDA-MB-231 and SKBr3, with a greater effect in SKBr3 cells, indicating mitotic arrest [41]. Nar has a higher binding affinity for bovine serum albumin (BSA) than VOnar, indicating that the larger complex of VOnar may affect the binding affinity and transport of VOnar within serum; however, the potential bioavailability needs to be further investigated [41]. Overall, these results indicate the potential of VOnar as a treatment for breast cancer. Nonetheless, further investigation is needed.

MCF-7 cells treated with Nar or Nar-Oxime (NarOx) decreased cell viability in a dose-dependent manner, while sparing normal L-929 mouse fibroblast cells with greater effects in NarOx-treated cells [42]. Additionally, both Nar and NarOx increased intracellular ROS and DNA damage in MCF-7 breast cancer cells [42]. Apoptosis was visually characterized using AO/EB staining, and NarOx-treated cells had increased apoptotic cell counts [42]. Overall, these data indicate that NarOx may increase the ability of Nar to decrease cell viability and increase intracellular ROS, DNA damage, and apoptosis of MCF-7 breast cancer cells.

Oxime derivatives of racemic Naringenin were synthesized, purified, and characterized using column chromatography [43]. Concentrations of 25 and 50 µM of each derivative were selected to be used in the MTT assay [43]. *Tert*-butyl substituted derivative (*tert*-butyl-Nar) had the most potent decrease in cell viability in MCF-7 and MDA-MB-231 breast cancer cells compared to any other compound tested and was comparable to Cisplatin, an established chemotherapeutic [43]. The benzyl-derivative (benzyl-Nar) exerted moderate growth inhibition in only the MCF-7 cell line, but the growth inhibitor effects were much lower than those of cisplatin [43], whereas Nar at these concentrations did not exert growth inhibitory effects against either cell line [43]. Flow cytometry cell cycle analysis revealed that treatment with *tert*-butyl-Nar resulted in increased proportion of cells in the G1 phase and G2/M phase and decreased S phase in MCF-7 cells, indicating cell cycle arrest, whereas in MDA-MB-231, no significant change was observed [43]. These data indicate the increased potential anti-breast cancer activity of Nar with the addition of a *tert*-butyl group compared to Nar alone; however, further investigation into the potential antitumor activity and the mechanism of action of the *tert*-butyl derivative is required.

Treatment of MCF-7 breast cancer cells with Nar and Nar cyclic aminoethyl derivatives (NDs) (4-methylpiperidine, piperidine, morpholine, and pyrrolidine) decreased cell viability, whereas treatment of healthy brain astrocyte C8-D1A cells was significantly less affected [44]. Furthermore, PCR analysis revealed increased mRNA levels of p53, caspase 3, and Bax in MCF-7 cells treated with Nar and NDs, with no change in Bcl2 mRNA levels in ND treated MCF-7 cells but an increase in Nar-treated MCF-7 cells [44]. Treatment with piperidine, pyrrolidine, and Nar increased Cyt-C mRNA, a pro-apoptotic protein; however, treatment with 4-methylpiperidine and morpholine resulted in no change [44]. Furthermore, treatment with NDs but not Nar resulted in increased APAF1 mRNA levels, a pro-apoptotic protein [44], whereas in healthy astrocyte cells, no change in the mRNA levels was seen, except for slight increases with Nar treatment [44]. Overall, these results indicate that Nar and NDs act through the mitochondrial apoptosis signaling pathway to decrease cell viability in MCF-7 breast cancer cells while sparing normal healthy astrocyte cells.

Treatment of MCF-7 cells with nanosuspension of Naringenin (NarNS) resulted in a greater decrease in cell viability than treatment with Nar alone [45]. Additionally, treatment with NarNS resulted in a significant increase in intracellular ROS compared to Nar-treated cells and increased lipid peroxidation, indicating that oxidative stress may be responsible for the greater decrease in cell viability with NarNS treatment [45]. Glutathione (GSH) is responsible for maintaining cellular oxidation-reduction homeostasis; therefore, alterations can indicate functional damage to a cell. NarNS treatment of MCF-7 cells decreased GSH levels, further indicating oxidative stress [45]. Additionally, NarNS treatment decreased mitochondrial membrane potential (MMP) and increased caspase 3 activity in MCF-7 cells, indicating increased apoptosis. NarNS also significantly increased apoptotic morphological changes, such as increased condensed or fragmented chromatin [45]. Overall, these results indicate that NarNS may increase the ability of Nar to treat breast cancer and may potentially be due to better delivery and a sustained release.

Curcumin–Naringenin–D-coated magnetite nanoparticles (CUR-NAR-D-MNPs) were prepared and characterized. Treatment of MCF-7 breast cancer cells with CUR-NAR-D-MNPs decreased cell viability, and immunofluorescence revealed that treatment significantly increased apoptotic (early and late-stage) and necrotic cells [46]. Additionally, 48 h incubation of MCF-7 cells with CUR-NAR-D-MNPs before radiotherapy (RT) increased apoptosis and necrosis of MCF-7 cells compared to RT or CUR-NAR-D-MNP alone [46]. Post CUR-NAR-D-MNP treatment ROS levels are significantly increased in MCF-7 cells, with an even greater increase in ROS in CUR-NAR-D-MNP-treated cells exposed to RT [46]. Therefore, CUR-NAR-D-MNP has a radiosensitizing effect in MCF-7 breast cancer cells and decreases cell viability and increases apoptosis, indicating its therapeutic potential as a breast cancer treatment.

Naringenin-loaded poly lactic-coglycolic acid (PLGA)-doxorubicin nanoparticles (PDNG NPs) were synthesized, characterized, and drug release analyzed, revealing Nar and DOX were released in a similar manner, with Nar being released slightly quicker than DOX [47]. Treatment of MDA-MB-231, MCF-7, and T47D breast cancer cells with Nar-loaded PLGA NPs significantly decreased cell viability compared to DOX, Nar, or PDNG NPs alone, with the greatest decrease in cell viability in MDA-MB-231 cells [47]. Furthermore, treatment of HBL-100 human non-cancerous breast cells and H9c2 non-cancerous rat myoblasts with Nar-loaded PDNG NPs only decreased cell viability at very high concentrations, indicating the selective toxicity to cancerous cells [47]. Additionally, Nar-loaded PDNG NP treatment of MDA-MB-231 cells increased intracellular ROS and increased mitochondrial membrane depolarization, indicating mitochondrial-dependent cell death [47]. Flow cytometry analysis revealed increased apoptosis and G1 and G2/M phase arrest [47]. Confocal microscopy revealed Nar-loaded PDNG NPs penetrated the cell membrane and could enter the nucleus after 24 h; however, these were not compared to a control or the other compounds alone [47]. Additionally, Western blot analysis revealed increased caspase 3 and 9 protein levels; however, these were not the active forms [47]. Overall, these data indicate the potential of Nar-loaded PDNG NPs to selectively decrease cell viability of breast cancer cells by increasing apoptosis via increasing intracellular ROS and mitochondrial membrane depolarization; however, further research is needed to confirm these results.

Smart polymeric nanoparticles loaded with 0.3 mg/mL Nar (NarSPNP) were prepared to be pH and thermosensitive. MCF-7 cells treated with NarSPNP decreased cell viability and, compared to Nar alone, had lower IC50 values with no effect in normal human dermal fibroblast cells (HDFa) [48]. Flow cytometry analysis indicated NarSPNP treatment increased early and late apoptosis compared to Nar alone, with no effect in HDFa cells [48]. Additionally, NarSPNP treatment increased the ratio of MCF-7 cells in the G1 phase and decreased the ratio of cells in the S phase and G2, indicating cell cycle arrest [48]. Overall, these data indicate that NarSPNP is more effective than Nar alone in inhibiting MCF-7 breast cancer cell growth.

The *Saccharomyces cerevisiae* BJ5464-NpgA yeast strain was used to host plasmids expressing two O-methyltransferases, HsOMT and LtOMT, from *Homo sapiens* and *Litsea cubeba*; Nar was added into the media, and methyl groups were added to the hydroxyl groups on Nar [49]. The methyl–Nar was extracted using ethyl acetate and characterized and purified using HPLC; one product was obtained, 7-methoxyl-Nar, catalyzed by HsOMT, and contained one additional methyl group than Nar, located at C-7. In MCF-7 breast cancer cells, treatment with 7-methoxyl–Nar significantly decreased cell proliferation and increased cytotoxicity compared to Nar. These results indicate that the addition of a methyl group to Nar may increase its anti-proliferative and cytotoxic effects in breast cancer; however, further investigation is needed into the mechanisms behind these effects.

Treatment of MCF-7 with polyethylene glycol-modified albumin nanoparticles loaded with Nar (Nar-NPs) resulted in decreased cell viability, whereas in normal human fibroblast cells (HFFs), there was no change in cell viability [50]. Additionally, treatment of MCF-7 breast cancer cells with Nar-NP resulted in cell cycle arrest in the subG1 phase and increased caspase 3, 8, and 9 mRNA levels, indicating increased apoptosis [50]. These results indicate the potential of Nar-NPs to decrease cell viability and increase apoptosis of breast cancer cells while sparing normal healthy cells.

MDA-MB-231 cells treated with Nar or chitosan-encapsulated Naringenin (CS-NPs/NAR) had decreased cell viability and proliferation, with CS-NPs/NAR having the greatest effect [51]. Additionally, both CS-NPs/NAR and Nar treatment increased nitrate, xanthine oxidase (XOD), and ROS, while decreasing xanthine dehydrogenase (XDH) levels, indicating increased oxidative stress; however, the greatest effect was seen with CS-NPs/NAR treatment [51]. Furthermore, CS-NPs/NAR treatment increased caspase-3 protein levels and increased DNA fragmentation, indicating increased apoptosis, which was further assessed through flow cytometry, which then revealed increased apoptosis in both treatment groups [51]. These results indicate CS-NPs/NAR treatment increases oxidative stress and activation of caspase-3, which may lead to increased apoptosis and reduced proliferation of breast cancer cells.

Treatment of MDA-MB-231 and MCF-7 cells with Naringenin-7-O-glucoside (Nar-Glucoside) decreased cell viability; however, there was a greater effect in the MDA-MB-231 TNBC cell line than in the MCF-7 luminal A cell line [52]. Additionally, Western blot analysis revealed that treatment of MDA-MB-231 cells with Nar-Glucoside increased DNA fragmentation, a marker of apoptosis. Additionally, both PCR and Western blot analysis revealed decreased epidermal growth factor receptor (EGFR) levels [52]. EGFR is important in cell growth, proliferation, and differentiation, and is, therefore, an attractive target to decrease breast cancer proliferation [52]. However, the study does not investigate the molecular mechanisms involved in the downstream process of DNA fragmentation or EGFR [52]. Overall, this study provides preliminary evidence investigating the anti-breast cancer potential of Nar-Glucoside to decrease cell viability through increased DNA fragmentation and decreased EGFR levels in breast cancer cells.

In MDA-MB-231 triple-negative breast cancer cells, treatment with Nar and liposome-encapsulated Nar (Lip-Nar) significantly decreased cell viability; however, decreased cell viability was seen at lower concentrations of Lip-Nar compared to Nar. At the same concentrations of Nar and Lip-Nar, no significant decrease in cell viability was seen when normal human keratinocyte cells (HaCaT) [53] were used, indicating the potential of Lip-Nar to specifically decrease cell viability of TNBC cells in vitro; however, additional investigation is required into the potential mechanisms behind these effects.

Evidence indicates that Nar and its derivatives exert anti-breast cancer effects primarily by inducing apoptosis and reducing cell viability and proliferation. Nanosuspensions and nanoparticle-encapsulated forms of Nar increase ROS, decrease mitochondrial membrane potential, and elevate caspase-3 and caspase-9 activity, promoting mitochondrial-mediated apoptosis and DNA fragmentation in breast cancer cells [45,47,50,51]. Nar derivatives also upregulate pro-apoptotic genes such as p53, Bax, caspase-3, and cytochrome-c, further supporting activation of intrinsic apoptotic pathways [44]. In addition, Nar and its modified analogs reduce breast cancer cell proliferation and viability, with some derivatives demonstrating greater cytotoxic and cell cycle arrest effects than Nar alone [43,49]. Importantly, several studies report selective toxicity toward breast cancer cells with minimal effects on normal cells [44,47,50,53]. Collectively, these findings suggest that chemical modification or nanoparticle delivery of Nar enhances its anti-breast cancer efficacy by promoting oxidative stress–mediated apoptosis and inhibiting breast cancer cell growth.

## 4. Naringenin in Combination with Chemotherapy: In Vitro

Studies that have examined the effect of Nar in combination with chemotherapy in cultured breast cancer cells are presented below and in Table 3. Chemotherapy is a standard treatment for breast cancer that uses combinations of cytotoxic drugs, such as tamoxifen, cyclophosphamide, and doxorubicin, to destroy or inhibit the growth of cancer cells. It may be administered before surgery to shrink tumors and reduce lymph node involvement or after surgery to lower the risk of recurrence [3]. However, chemotherapy has several limitations, including systemic toxicity, negative impacts on patient quality of life, and the development of treatment resistance in some breast cancer cells, which can reduce therapeutic effectiveness and contribute to disease recurrence [3].

In MCF-7 breast cancer cells grown in charcoal-stripped fetal bovine serum (CS-FBS) medium, Nar treatment decreased proliferation, and treatment in combination with the established chemotherapeutic tamoxifen (Tam), a selective estrogen receptor modulator, resulted in a greater decrease in cell proliferation than either treatment alone [54]. Similarly, flow cytometry revealed Nar decreased cell viability and induced cell death. Notably, when combinations of Nar and Tam were used, lower concentrations could achieve the same effect as either alone, indicating synergy [54]. Furthermore, Nar alone and in combination with Tam increased apoptosis, decreased total protein levels of caspase 7, and increased PARP protein levels [54]. However, the cleaved, active form of caspase 7 and PARP was not investigated [54]. Western blot analysis revealed Nar and combination treatment decreased total levels of ERK1/2 and Akt with no effect on phosphorylated levels, whereas Tam alone did not affect ERK1/2 or Akt expression [54]. Nar treatment increased cytosolic levels of estrogen receptor alpha (ERα), whereas combination treatment resulted in an even distribution of ERα between nuclear and cytosolic fractions [54]. Overall, the combination treatment of MCF-7 breast cancer cells with Nar decreased proliferation and increased apoptosis, and was found to act synergistically with Tam.

Treatment of human MDA-MB-231 breast cancer cells with Nar resulted in a dose-dependent decrease in cell viability [55]. Flow cytometry analysis revealed Nar treatment increased early and late-stage apoptosis, further indicated by increased caspase 3 and 8 activity. Combination treatment with Nar and cyclophosphamide (cpm), an established chemotherapeutic, resulted in a greater decrease in cell viability, a greater increase in early and late-stage apoptosis, and a greater increase in caspase 3 and 8 activity than either treatment alone. Additionally, the use of IL-6 as a positive control to inhibit apoptosis resulted in increased antiapoptotic protein B-cell lymphoma 2 (Bcl-2) mRNA, decreased proapoptotic protein Bcl-2-associated X protein (Bax) mRNA, and increased phosphorylated Signal Transducer and Activator of Transcription 3 (STAT3) protein levels. In silico analysis revealed a good theoretical affinity of Nar to bind to the pTyr705 site on the SH2 domain of STAT3, indicating the potential of Nar to act as a STAT3 antagonist. As predicated, Nar treatment alone partially attenuated the IL-6 effects and resulted in decreased Bcl-2 mRNA, increased Bax mRNA, and decreased phosphorylated STAT3 protein levels. Importantly, combination treatment with Nar and cpm resulted in greater effects and significantly decreased Janus Activated Kinase 2 (JAK2) and STAT3 mRNA levels, which were not seen in any individual treatments. This study provides strong evidence that combination treatment of MDA-MB-231 breast cancer cells with Nar and cpm results in greater induction of apoptosis than either compound alone, even in the presence of IL-6, a potent anti-apoptotic cytokine. Overall, indicating the potential of Nar to be used as a chemosensitizing agent.

The combination treatment of MDA-MB-231 breast cancer cells with Nar and doxorubicin (DOX), a common chemotherapeutic, resulted in a significant decrease in cell viability, with an even greater decrease in cell viability in cells treated with Nar, DOX, and metformin (Met) [56]. However, treatment of 4T1 murine TNBC cells with Nar and doxorubicin did not result in any significant change in cell viability; nonetheless, treatment with Nar, DOX, and Met significantly decreased cell viability [56]. Further research is needed into the mechanisms behind this; however, overall, these results indicate the potential anti-breast cancer effects of combination treatment.

Nar treatment alone of HCC1806 (CRL-2335), a TNBC cell line, resulted in no difference in cell viability; however, it decreased the IC50 of paclitaxel (PTX), an established chemotherapeutic, indicating the potential chemo-sensitizing effects of Nar [57]. The NR3C2 gene was identified through The Cancer Genome Atlas Program (TCGA) Data Set as playing an important role in the overall survival of individuals with TNBC, with higher expression indicating a higher overall survival rate in those with TNBC [57]. In 4T1 and HCC1806 TNBC cell lines with siRNA knockdown of NR3C2, Nar treatment had no effect on the IC50 values of PTX [57]. Overall, Naringenin may target the NR3C2 gene to enhance the efficacy of PTX in TNBC cell lines.

There is limited evidence that chemotherapeutic agents in combination with Nar demonstrate anti-breast cancer activity by promoting apoptosis and reducing cell proliferation and viability [54,55,56,57]. Nar, in combination with cpm, increases early and late apoptosis and elevates caspase-3 and caspase-8 activity in MDA-MB-231 cells, while decreasing Bcl-2, increasing Bax, and inhibiting JAK2/STAT3 signaling, thereby counteracting IL-6–mediated anti-apoptotic effects compared to cpm alone [55]. Nar, in combination with chemotherapeutics, also decreases proliferation and viability in MCF-7 and MDA-MB-231 cells compared to the chemotherapeutic alone and enhances the cytotoxic effects of chemotherapeutics such as tamoxifen, cyclophosphamide, and doxorubicin, partly through reduced ERK1/2 and Akt expression and altered ERα localization [54,55,56].

## 5. Naringenin in Combination with Radiotherapy: In Vitro

Studies that have examined the effect of Nar in combination with radiotherapy in cultured breast cancer cells are presented below and in Table 4. Radiation therapy (RT) is a key component of breast cancer management and is commonly administered following surgery to reduce tumor burden and lower the risk of local recurrence [3]. RT is typically delivered several weeks after surgery or chemotherapy and is often administered in fractionated doses, which allow normal tissues to repair DNA damage while maintaining tumor radiosensitivity [3]. However, limitations of RT include potential toxicity to surrounding healthy tissues, the risk of tumor cell repopulation during prolonged treatment intervals, and the development of radioresistance, which may ultimately reduce therapeutic efficacy and contribute to disease recurrence [3].

4T1 breast cancer cells treated with Nar had decreased PKC-ζ phosphorylation with no change in PKC-ζ mRNA levels; PKC-ζ phosphorylation is associated with increased transforming growth factor beta 1 (TGF-β1) secretion, which increased Treg differentiation, an immune cell that acts as an immunosuppressant and inhibits the ability of the immune system to recognize and kill cancer cells [58]. Nar significantly decreased secretion of extracellular vesicles and TGF-β1 secretion even in the presence of radiation [58]. Additionally, treatment of naïve spleen cells with TGF-β1 isolated from 4T1 breast cancer cells treated with Nar alone or treated with Nar in combination with radiation significantly decreased Treg differentiation from CD4+ T cells [58]. Overall, these data indicate that Nar and prevent radiation induced Treg differentiation, TGF-β1secretion, and PKC-ζ phosphorylation, overall potentially contributing to the ability of Nar to decrease breast cancer proliferation. Additionally, exposure of 4T1 cells to radiation significantly increased superoxide radicals (O2˙−) and increased zinc release; zinc increases PKC-ζ phosphorylation, and when cells were additionally treated with Nar, these effects were inhibited [58].

MDA-MB-231 breast cancer cells treated with Lip-Nar resulted in a significant decrease in cell viability compared to Nar. Additionally, MDA-MB-231 cells treated with radiation in combination with Nar or Lip-Nar significantly decreased cell viability compared to radiation alone, with a greater decrease in cell viability with Lip-Nar [53]. In HaCaT cells, there was a significantly lower decrease in cell viability in the Lip-Nar group in combination with radiation compared to the MDA-MB-231 cells with the same treatment [53]. Additionally, Nar combined with radiation significantly decreased colony formation, and Lip-Nar combined with radiation resulted in no colony formation, whereas HaCaT cells treated with Nar or Lip-Nar in combination with radiation resulted in a similar overall reduction in observable colonies between all treatments [53]. Collectively, these findings indicate the radio-sensitizing potential of liposome-encapsulated Nar; however, further research is needed to investigate the mechanisms behind these effects.

The limited evidence of radiotherapy in combination with Nar demonstrates potential radiosensitizing effects in breast cancer cells by reducing tumor cell survival and modulating immune signaling pathways [53,58]. Combination treatment with Nar decreases PKC-ζ phosphorylation and TGF-β1 secretion, which may limit regulatory T cell differentiation and enhance anti-tumor immune responses [58]. When combined with radiation, both Nar and LipNar significantly reduce breast cancer cell viability and colony formation, indicating enhanced radiosensitivity and suppression of clonogenic survival [53]. Despite these findings, only a limited number of studies have investigated the combined effects of Nar and radiation therapy, highlighting the need for further research in this area.

## 6. Naringenin in Combination with Hormone Therapy: In Vitro

Studies that have examined the effect of Nar in combination with hormone therapy in cultured breast cancer cells are presented below and in Table 5. Hormone therapy is used to inhibit the growth of hormone receptor-positive breast cancers by blocking estrogen signaling or reducing estrogen production, thereby limiting hormone-dependent tumor proliferation [3]. Adjuvant endocrine therapy (AET) is typically administered for 5–10 years in patients with non-metastatic hormone receptor-positive disease and includes treatment strategies such as selective estrogen receptor modulators, aromatase inhibitors, ovarian ablation, or ovarian function suppression [3]. Although AET significantly reduces recurrence and mortality rates, its effectiveness is limited to hormone receptor-positive tumors, and treatment may be constrained by long therapy duration, adverse effects, and the development of endocrine resistance, which can compromise long-term therapeutic outcomes [3].

MCF-7 ER+ breast cancer cells treated with Nar alone resulted in decreased cell viability, migration, and invasion, and combined treatment with Tamoxifen (Tam) resulted in an even greater decrease [59]. Matrix metalloproteinase 9 (MMP-9) and MMP-2, proteins associated with cell invasion, were analyzed using RT-qPCR; Nar alone resulted in a significant decrease in both MMP-2 and 9, with a greater decrease when treated in combination with Tam [59]. MCF-7 cells treated with Nar alone and in combination with Tam resulted in an increased proportion of cells in the S and G2/M phase of the cell cycle, indicating cell cycle arrest [59]. Furthermore, mRNA levels of key proteins involved in cell cycle regulation and tumor suppression—p53 and p21—were increased, while mRNA levels of cyclin E and cyclin D—cell cycle proteins involved in G1 and S phase—were significantly decreased in MCF-7 cells treated with Nar, with a greater effect in cells treated in combination with Tam [59]. Additionally, flow cytometry revealed Nar treatment increased the proportion of cells in early and late apoptosis after 48 h treatment, with a greater increase with combination treatment [59]. Hoechst nuclear staining in MCF-7 cells treated with Nar alone and in combination with Tam revealed nuclear condensation, further indicating apoptosis [59]. Furthermore, Nar treatment resulted in mitochondrial apoptosis-related protein Bcl-2, an anti-apoptotic protein, mRNA levels decreasing, and Bax, a pro-apoptotic protein, mRNA levels increasing [59]. Surprisingly, combination treatment significantly decreased both Bcl-2 and Bax mRNA levels. To further investigate the mechanism of apoptosis, intracellular ROS levels were measured; MCF-7 cells treated with Nar alone significantly increased intracellular ROS, with an even greater effect in combination-treated cells [59]. These results indicate intracellular ROS and mitochondrial dysfunction may be contributing to the apoptotic effects of Nar alone and in combination with Tam. RT-qPCR and Western blotting analysis revealed Nar treatment significantly increased mRNA and protein levels of nuclear estrogen receptors (nER), ERα66 and ERβ, and decreased mRNA and protein levels of membrane estrogen receptors (mER), ERα36 and GPR30 [59]. Interestingly, combination treatment with Nar and Tam resulted in decreased protein and mRNA levels of ERα66, ERα36, and GPR30 and increased ERβ mRNA and protein levels [59]. Overall, these results suggest that the combination treatment of ER+ breast cancer cells could inhibit cell proliferation more effectively than Nar alone.

Nar has shown potential to enhance the effects of hormone therapy in ER+ breast cancer cells. In MCF-7 cells, Nar, in combination with Tam compared to Nar alone, decreased cell viability, migration, and invasion and inhibited proliferation by inducing cell cycle arrest, decreasing MMP-2 and MMP-9 expression, and promoting apoptosis through increased ROS production and mitochondrial apoptotic signaling [59]. Nar also decreased ER surface expression, indicating a role in altering hormone responsiveness and contributing to the enhanced anti-tumor effects observed with Tam co-treatment [59]. However, these findings are currently based on a single in vitro study, highlighting the need for further in vitro and in vivo research to better evaluate the therapeutic potential of Nar in combination with hormone therapy.

## 7. Naringenin in Combination with Plant-Derived Antioxidants: In Vitro

Studies that have examined the effect of Nar in combination with other natural potential therapeutics in cultured breast cancer are presented below and in Table 6.

Treatment of MCF-7 breast cancer cells with Nar alone and in combination with Balsamin (Bal), a type 1 ribosome inactivating protein known to have antioxidant properties, significantly decreased cell viability [60]. Additionally, cells treated with Nar alone appeared to have apoptotic morphological changes, such as cell shrinkage, cell surface detachment, and increased cytoplasmic density; these effects were greater in the combination treatment. Nar alone increased caspase 3 and 9 activity in MCF-7 cells, and combination treatment resulted in an even greater increase. Furthermore, anti-apoptotic, Bcl-2, and Bcl-XL mRNA levels were significantly decreased, and tumor suppressors and pro-apoptotic, Bax, Bid, Bad, and p53 mRNA levels increased in both Nar and Nar with Bal-treated cells, with a greater effect in combination-treated cells. Treatment of MCF-7 breast cancer cells with Nar increased mRNA levels of CHOP and GRP78, and proteins involved in ER-stress-mediated apoptosis had a greater increase in combination treatment with Nar and Bal. Overall, these results indicate the potential of Nar and Bal combination treatment to increase the anti-breast cancer effects of Nar alone, such as decreasing cell viability and increasing mitochondrial and ER-mediated breast cancer cell apoptosis.

Treatment of MCF-7 breast cancer cells with Nar decreased cell viability, Bcl-2 mRNA levels, and mitochondrial membrane potential, indicating decreased proliferation and increased apoptosis [61]. Combination treatment of MCF-7 breast cancer cells with Nar and Quercetin (Que), another polyphenol, resulted in a synergistic effect and resulted in a larger decrease in cell viability, Bcl-2 mRNA levels, and mitochondrial membrane potential than Nar alone [61]. Combination treatment decreased caspase 3/7 activity, further indicating increased apoptosis [61]. Additionally, Nar treatment increased malondialdehyde, a product of lipid peroxidation, indicating increased lipid degradation and oxidative stress [61]. Overall, these results indicate the synergistic effects of Nar and Que to decrease breast cancer cell viability, proliferation, and increase apoptosis and cellular stress.

In MCF-7 and MDA-MB-231 breast cancer cells, treatment with Nar alone significantly decreased cell viability and migration, and had a greater decrease when treated in combination with quercetin (Que) or fisetin (Fis), two other flavonoids shown to have anticarcinogenic properties [62]. PCR analysis revealed increased caspase 3, 8, and 9 mRNA levels and a decrease in Bcl-2 mRNA levels in MCF-7 and MDA-MB-231 breast cancer cells treated with Nar alone, as well as a synergistic effect when treated in combination with Que/Fis, indicating increased apoptosis [62]. Additionally, Nar treatment of both cell lines increased miR-1275 mRNA levels, a tumor suppressor miRNA, and decreased mir-27a-3p mRNA levels, an oncogenic miRNA, with a greater effect in combination treatment [62]. These data indicate the synergistic effects of Nar in combination with Que or Fis to decrease cell viability and migration of breast cancer cells. Further investigation is needed to determine the mechanisms behind these effects.

Nar, in combination with antioxidant compounds, has demonstrated enhanced anti-breast cancer effects in vitro. In MCF-7 and MDA-MB-231 breast cancer cells, Nar combined with antioxidants such as balsamin, quercetin, or fisetin resulted in greater reductions in cell viability, proliferation, and migration compared with Nar alone [60,61,62]. However, these findings are limited to a small number of in vitro studies, and future research should focus on elucidating the underlying molecular mechanisms and validating these effects in further in vitro and in vivo models.

## 8. Naringenin Against Breast Cancer: In Vivo Animal Studies

Studies that have examined the effect of Nar administration in mice with breast cancer cells are presented below and in Table 7.

In 4T1 breast cancer cells treated with Nar, there was no change in cell viability. Additionally, in female mice with 4T1 breast cancer xenografts, Nar treatment did not decrease tumor size, indicating that Nar does not have a direct effect on decreasing tumor cell viability [63]. However, in female mice with resected 4T1 xenografts, post-resection oral administration of Nar significantly decreased lung metastatic colonies and increased survival [63]. Additionally, oral administration of Nar increased activated CD4+ and CD8+ T cells [63]. Expressing interferon-γ (IFN-γ) and IL-2 in CD8+ T lymphocytes is critical for tumor eradication. In tumor-resected mice, Nar treatment increased CD8+ T cells expressing IFN-γ and IL-2 to comparable levels to the control [63]. Overall, these results indicate that Nar promotes the activation and antitumor function of T cells in tumor-resected mice [63]. In vitro investigation of naive splenic T cells treated with Nar revealed decreased TGF-β1 and IL-10 expressing CD4+ and CD8+ T cells, indicating decreased immunosuppressive cytokines in T cells [63]. Additionally, Nar treatment of naive splenic T cells decreased the amount of regulatory T cells (Tregs); Tregs suppress the normal immune response, and, therefore, these results indicate that Nar treatment restores immune balance [63]. Overall, these results indicate that Nar has the potential to restore immune cell balance and decrease metastasis post tumor resection.

In vitro investigation revealed Nar treatment of MCF-7 breast cancer cells stably transfected with human CYP19 (MCF-7aro) significantly decreased aromatase activity [64]. In ovariectomized female athymic mice transplanted with MCF-7aro breast cancer cells and administered androstenedione injection (AD), a precursor to estrogen and testosterone, Nar treatment had no effect on tumor growth, body weight, tumor weight, and liver weight [64]. Overall, these data indicate Nar does not decrease the tumor growth of MCF-7aro breast cancer cells in mice.

In vitro data indicate that Nar reduces TGF-β1 trafficking and secretion through decreasing PKC phosphorylation in 4T1-Luc2 breast cancer cells transfected to overexpress TGF-β1 (4T1-TGF-β1). There is an association between elevated transforming growth factor (TGF)-β1 levels and increased breast cancer metastasis; TGF-β1 promotes metastasis by transforming T cells into regulatory T cells (Tregs), which facilitates the evasion of cancer cells from the host immune cells. Female Balb/c mice, xenografted with 4T1-TGF-β1 breast cancer cells and treated with Nar, had decreased pulmonary metastasis and prolonged survival, with no change in primary tumor size compared to control [25]. Additionally, compared to mice treated with a 1D11 antibody, a TGF-β1 neutralizing antibody, Nar-treated mice had prolonged survival and a greater decrease in metastasis [25]. Furthermore, Nar treatment significantly decreased activated TGF-β1 in the tissue (tumor, lung, and spleen) and serum of mice, which may account for the decreased metastasis [25]. Elevated TGF-β1 increases Foxp3 expression, which induces the differentiation of T cells into Tregs and is the master regulator that mediates the immunosuppressive function of Tregs; Nar treatment of mice significantly decreased Foxp3 levels in the lungs and serum compared to control mice, indicating decreased Treg differentiation due to decreased TGF-β1 levels [25]. Additionally, Nar treatment decreased CD4+ CD25+ Foxp3+ Tregs in the lungs and CD103+ CD4+ Foxp3+ Tregs in the spleen of 4T1/TGF-β1 tumor-bearing mice, further confirming decreased Tregs differentiation [25]. Additionally, in Nar-treated mice, the TGF-β1 increase in CD4+ Gr11b+ T cells—immune cells associated with immunosuppression—and the decrease in activated CD4+ CD44+ CD62L– T cells—immune cells associated with immune cell recruitment and potentially direct tumor apoptosis—in the lungs and spleen were abolished [25]. Furthermore, TGF-β1 decreased CD8+ IFNγ+ T cells, an immune cell responsible for killing tumor cells and immune cell recruitment, in the spleen, which was attenuated in Nar-treated mice [25]. In the lungs of Nar-treated mice, mRNA levels of IFNγ and granzyme-B, two effector molecules that induce apoptosis of tumor cells, were significantly increased [25]. Nude mice with 4T1/TGF-β1 xenografts were given T cells transferred from either tumor-bearing mice who had no treatment or Nar treatment; the nude mice with Nar-treated T cells had significantly decreased incidence of pulmonary metastasis and whole-body metastasis [25]. Overall, these data indicate that Nar attenuates the TGF-β1 induced immunosuppressive environment that promotes metastasis of breast cancer in tumor bearing mice and therefore may be an attractive target to decrease breast cancer metastasis.

Ovariectomized C57BL/6 mice injected with E0771 breast cancer cells, fed a high-fat diet (HFD) with high Nar (HN) (HFD + HN), compared to HFD with low-Nar (LN) (HFD + LN)-fed mice, had decreased body weight and calorie intake, lower blood glucose levels, and no difference in fasted blood insulin levels [26]. HFD + HN mice had increased concentrations of Nar in the serum and tissue (mammary adipose tissue and tumor tissue) compared to HFD + LN-fed mice. Importantly, as increased adipose mass promotes breast cancer tumor development and growth, HFD + HN-fed mice had decreased mammary, perigonadal, and mesenteric adipose mass while maintaining muscle mass compared to HFD mice. Individual adipocyte size was significantly decreased in HFD + HN-fed mice compared to both HFD + LN and HFD-fed mice. HFD + HN-fed mice had decreased monocyte chemoattractant protein-1 (MCP-1) mRNA levels in perigonadal and mammary adipose tissue and decreased IL-6 mRNA levels in perigonadal adipose tissue, indicating Nar decreases adipose mass and inflammatory adipokines even in the presence of an HFD. There was no difference in size or weight of mice in different treatment groups, and there was no difference in p-AMPK, p-p70 S6K, or CyclinD1 protein levels in the mammary tumors between HFD + HN, HFD + LN, and HFD-fed mice. However, HFD + HN-fed mice had increased Akt phosphorylation and mTOR phosphorylation compared to HFD-fed mice. Overall, these data indicate that oral administration of Nar was bioavailable and able to travel to target tissues but was not able to prevent tumor growth.

Previous in vitro data indicate that Nar decreases MDA-MB-231 breast cancer cell viability and increases apoptosis through increased caspase-3/7 activity, DNA fragmentation, and reduced NF-kB DNA binding [28]. In female Wistar rats, intravenous injection of DMBA to induce breast cancer, low, medium, and high Nar treatment doses dependently decreased the amount of body weight gained, tumor incidence, tumor burden, and tumor volume [28]. High-dose Nar significantly decreased lymph node and pulmonary metastases. Nar treatments decreased proliferating cell nuclear antigen (PCNA)-positive cells, DNA fragmentation, and increased caspase 3 and 9 activity in a dose dependent manor, indicating increased apoptosis [28]. Furthermore, Nar treatment dose dependently increased Bax mRNA and decreased Bcl-xl and Bcl-2 mRNA in the cytoplasm of mammary gland tumor tissues, further indicating increased apoptosis [28]. Nar treatment also dose dependently decreased mitochondrial-mediated apoptosis markers Apaf-1, voltage-dependent anion channel (VDAC), and cytochrome c mRNA while increasing procaspase-9 mRNA, indicating a return towards control levels [28]. Additionally, Nar treatment dose dependently decreased thiobarbituric acid-reactive substances (TBARSs), SOD, catalase, protein carbonyl, and nitrate levels while increasing GSH, vitamin C, vitamin E, and glutathione reductase levels, indicating increased antioxidant and decreased pro-oxidant response; moderate levels of ROS contribute to cancer growth, and, therefore, decreasing ROS may inhibit cancer progression [28]. Overall, these data provide evidence that orally administered Nar may dose dependently decrease tumor growth and development in rats. Further investigation is needed to confirm these results in other animals.

In vitro data indicate that Nar may decrease ER+ breast cancer cell growth and mammosphere formation by preventing ER mediated cell growth and survival through the activation of Death-associated factor 6 (DAXX) and inhibition of NOTCH signaling. DAXX plays an important role in inhibiting tumor-initiating cells (TICs). To further investigate these effects, an ovariectomized mouse xenograft model was developed, where mice were xenografted with either normal MCF-7 breast cancer cells or MCF-7 cells transfected with DAXX siRNA knockout (KO). Nar treatment in MCF-7 control mice decreased tumor incidence and size, whereas in DAXX KO MCF-7 tumor-bearing mice, Nar had no effect [29]. Additionally, in female athymic nude ovariectomized mice with an ER+ patient-derived xenograft (PDX) tumor, Nar treatment decreased tumor size compared to control, and immunoblotting revealed Nar increased DAXX protein levels and decreased NOTCH4 protein levels [29]. These data indicate that Nar increases DAXX to inhibit tumor development and growth. Additionally, Nar decreased mammosphere-forming efficiency (MFE), indicating decreased TIC survival [29]. However, in ovariectomized female athymic nude mice treated with E2 in combination with Nar or with Nar alone, there was no change in tumor growth, indicating that Nar had the potential to compete with and block estrogen effects to increase ER+ breast cancer growth [29]. Overall, these results indicate the potential of Nar to increase DAXX protein levels and decrease TICs, thereby decreasing tumor growth.

Combination treatment of MDA-MB-231 and 4T1 breast cancer cells with Nar, doxorubicin, and metformin decreases cell viability [56]. In mice with MDA-MB-231 breast cancer cell xenografts, Nar treatment in combination with metformin and liposomal doxorubicin (lip-dox) significantly decreased tumor volume compared to lip-dox + Nar and lip-dox alone [56]. This suggests a synergistic effect of Nar, Met, and lip-dox to decrease tumor size [56]. There was no significant change in body weight, organ weight, or blood glucose levels in any of the treatment groups [56]. Interestingly, lip-dox treatment significantly increased serum concentrations of TNF-α and IL-1β; however, combination treatment with Met and Nar significantly decreased these levels, and these data potentially indicate that Nar and Met may decreased overall toxicity of lip-dox treatment [56]. Hematoxylin and eosin (H&E) staining revealed increased necrotic area, and immunohistochemistry revealed decreased Ki-67, a cell proliferation marker, in combination-treated tumors compared to all other treatment groups, with no myocardial abnormalities [56]. Overall, these data indicate increased effectiveness and reduced toxicity of combination treatment.

MCF-7 breast cancer cells treated with CUR-NAR-D-MNP had increased radiosensitivity, as indicated by increased apoptosis and necrosis compared to radiation treatment alone [46]. Female Sprague–Dawley rats induced with breast cancer via oral ingestion of DMBA, treated with CUR-NAR-D-MNPs, showed no significant changes in liver or kidney histology or biochemical parameters compared to control; however, CUR-NAR-D-MNP-treated rats had increased GSH and CAT serum concentrations, indicating decreased oxidative stress [46]. CUR-NAR-D-MNP-treated rats had increased body weight compared to the tumor group, decreased tumor size, and increased necrosis and apoptosis of tumor cells, indicated by increased pyknotic nuclei, large eosinophilic granular areas, apoptotic bodies, and cellular debris [46]. Furthermore, CUR-NAR-D-MNP treatment increased the proportion of cells in the G0/1 phase, Bax, and caspase-3 levels while decreasing Bcl-2 levels in tumor cells, further indicating increased apoptosis [46]. Additionally, compared to control and radiation, CUR-NAR-D-MNP treatment alone or in combination with radiation increased P53, P21, and ROS levels while decreasing TNF-α and CD44 levels [46]. Overall, these data indicate that the anti-cancer and radio-sensitizing effects of CUR-NAR-D-MNP decreased breast cancer tumor growth.

In vitro data indicate Nar decreases aromatase activity and ER+ breast cancer cell viability. To further investigate the estrogenic potential of Nar, immature female Swiss albino mice were treated with Nar and revealed slightly delayed vaginal opening and cornification, indicating the absence of estrogenic activity [31]. Additionally, Nar, in combination with estrogen in immature mice, delayed vaginal opening, reduced uterine weight, and partially prevented vaginal cornification [31]. Importantly, no mortalities, morphological changes in the livers, or body weight changes were observed in Nar-treated groups [31]. Additionally, in an Ehrlich ascites carcinoma (EAC) tumor model in mature female Swiss albino mice, Nar treatment significantly decreased tumor size and aromatase activity [31]. These data indicate the partial anti-estrogen and antitumor activity of Nar to treat mammary tumors.

In vitro evidence indicates that Nar-loaded PDNG NPs decreased breast cancer cell viability and increased apoptosis while sparing normal cells. In Sprague–Dawley rats, an IV bolus dose of PDNG NP had increased half-life and decreased renal excretion compared to Nar or doxorubicin alone, indicating better bioavailability [47]. Additionally, PDNG NP treatment had better organ distribution than either compound alone; these data suggest a higher resistance time of DOX and Nar when encapsulated in PDNG NP [47]. In female nude mice injected with MCF-7 breast cancer cells to form tumors, PDNG NP treatment significantly decreased tumor size compared to individual treatments alone and control [47]. Additionally, mice treated with PDNG NP had decreased body weight, which may be an indication of better anti-tumor activity; however, no body composition analysis was performed [47]. These results indicate better bioavailability, distribution, and anti-breast cancer tumor activity of Nar and DOX in PDNG NP than either treatment alone; however, further investigation into the mechanisms behind these effects is required.

In female rats with methylnitrosourea (MNU)-induced breast cancer, the combination treatment of Nar, Met, and DOX had the greatest decrease in tumor incidence, number, multiplicity, and weight [65]. Nar alone did not significantly decrease any of these measurements; however, the combination of Nar and DOX decreased tumor incidence, number, and weight [65]. In a 4T1 xenograft breast cancer model in mice, Nar treatment did not decrease tumor weight; however, combination treatment with Nar + DOX decreased tumor weight and had moderate antitumor activity, and the greatest decrease in tumor size and anti-tumor activity was achieved with Nar + Met + DOX treatment [65]. Additionally, the combination treatment with Nar + Met + DOX had a comparable decrease in tumor size to a higher dose of DOX alone but had an increased survival rate of mice [65]. Total WBC and neutrophil counts were significantly decreased compared to the control in combination treatment; however, they were significantly higher than the higher dose of DOX, which produces a comparable decrease in tumor weight, indicating the combination treatment minimizes toxicity while maintaining efficacy [65]. In both the mouse and rat models, combination treatment significantly increased the tumor necrotic area [65]. Overall, these data indicate a synergistic effect of Nar + Met + DOX treatment against breast cancer.

Previous data in luminal A subtype and basal-like subtype breast cancer cell lines revealed Nar treatment decreases cell proliferation and colony formation by decreasing FKBP4 protein levels and increasing NRF2 protein levels [33]. In mice xenografted with T47D, Nar treatment significantly decreased tumor size and weight compared to the control [33]. However, in mice xenografted with T47D breast cancer cells with silenced NRF2 (T47D shNRF2), there was a significant increase in tumor size even in the presence of Nar, and tumor size was comparable to that of mice receiving no treatment [33]. Overall, these results indicate that the ability of Nar to decrease tumor size and weight is dependent on NRF2. Further investigation into the molecular mechanisms and bioavailability of Nar is required.

Female mice with explants of spontaneous mouse mammary tumor (SMMT), an invasive ductal carcinoma, developed spontaneously in female Balb/c mice, then treated with Nar alone had decreased tumor size compared to the control [66]. Tumor-bearing mice treated with Nar in combination with cryptotanshinone (CPT), a naturally occurring compound found within sage, had a greater decrease in tumor size than Nar alone [66]. Splenocytes isolated from experimental mice that were treated with Nar alone or in combination with CPT had increased proliferation, with a greater increase in combination treatment, indicating increased immune cell proliferation [66]. Additionally, splenocytes from Nar-treated mice had decreased IL-4 secretion, a cytokine that promotes proliferation and survival of tumor cells, with a greater effect in combination-treated mice splenocytes; only combination-treated mice splenocytes had increased IFN-γ secretion, a cytokine with anti-tumor activity, indicating a potential shift in the immune system to decrease tumor growth [66]. Splenocytes from Nar-treated mice had decreased splenic CD4+ CD25+ Foxp3+ T cells but had no difference in tumor CD4+ CD25+ Foxp3+ T cells; however, combination treatment splenocytes had significantly decreased tumor and splenic CD4+ CD25+ Foxp3+ T cells. CD4+ CD25+ Foxp3+ T cells are Tregs, immune cells that suppress the activity of other immune cells therefor decreasing intratumor levels of Tregs may increase the body’s immune response to tumors [66]. Western blotting of tumor tissue revealed Nar-treated mice tumors had no change in JAK2 or STAT3 phosphorylation; however, CPT-treated tumors alone had decreased JAK2 and STAT3 phosphorylation, with a greater effect in combination-treated tumors [66]. PCR analysis of tumors indicated that the combination treatment, but not Nar alone, decreased Bcl-XL and Mcl-1 mRNA levels, two proteins downstream of JAK2 and STAT3, further confirming decreased JAK/STAT activation, overall indicating increased apoptosis [66]. Additionally, previous research had revealed that decreased JAK/STAT activation may decrease Foxp3 expression and functions of differentiated CD4 + CD25+ T lymphocytes, which may have contributed to the decreased CD4+ CD25+ Foxp3+ T cells in mice treated in combination with Nar and CPT [66]. Overall, these data indicate Nar combined with CPT may modulate the immune system to decrease breast cancer growth.

Female rats treated with DMBA to induce breast cancer, then treated with Nar, had decreased incidence, number, and delayed development of tumors [67]. Nar in combination with zinc did not decrease tumor incidence; however, tumor development was delayed, and tumor weight was decreased [67]. Additionally, urine samples indicated increased N6-methyl-2′-deoxyadenine and 3-methyladenine in the urine of rats treated with Zinc and Nar but not in rats treated with Nar alone [67]. Methylated compounds can be used as an indicator of whole-body turnover and degradation of methylated RNA, which regulates protein synthesis, increased levels as seen in rats treated with zinc, may indicate an increased neoplastic process [67]. Overall, these data indicate that Nar may decrease the development and progression of DMBA induced breast cancer in rats. Further information is required to investigate the molecular mechanisms of Nar to inhibit tumor formation.

In vitro data indicate that in human normal liver cells (LO2), Nar treatment can increase estrogen metabolism by increasing estrogen sulfotransferase (EST), HNF4α, and decreasing FXR protein levels, indicating its potential to inhibit ER+ breast cancer cell growth [38]. In zebrafish embryos, Nar treatment did not result in embryotoxicity or affect hatching and development processes even at the highest concentration used, indicating Nar is safe [38]. Additionally, in a zebrafish DIL-stained E0771 breast cancer xenotransplantation model, Nar treatment decreased tumor growth and metastasis, and promoted the metabolism of estradiol into E2-3-O-sulfate in a dose-dependent manner, even in the presence of cortisol, which promotes breast cancer growth and metastasis through increased estradiol [38]. Furthermore, Nar treatment attenuated the increased FXR expression and decreased EST expression in the liver caused by cortisol [38]. Cortisol increases cholic acid levels, which is reversed by Nar and Nar inhibited cholic acid induced breast cancer growth and metastases in a dose dependent manor [38]. These data indicate that Nar could potentially decrease breast cancer growth and metastasis and modulate estradiol metabolism through the FXR/EST pathway in zebrafish. In mice with E0771 breast cancer cells xenografts and under chronic unpredictable mild stress (CUMS), which simulates depression and significantly increases breast cancer growth and development, Nar treatment increased mobility time and sucrose preference comparable to control mice, indicating decreased depression-like behavior [38]. Additionally, Nar treatment significantly decreased tumor volume, lung metastases, and metastasis-related protein levels (N-cadherin and vimentin) and decreased Ki67 protein levels in mice under CUMS [38]. In mice under CUMS, Nar treatment decreased the CUMS-induced increase in estradiol and increased estradiol-3-O-sulfate levels in the tumor, liver, and serum, and prevented CUMS induced increase in FXR and decrease in EST protein levels in the liver [38]. Overall, these findings confirm the zebrafish findings that Nar can attenuate CUMS induced breast cancer cell growth and metastases by increasing estradiol metabolism through the FXR/EST pathway.

In vitro evidence indicates that Nar attenuates radiation induced increases in superoxide radicals and size release, thereby decreasing PKC-ζ phosphorylation. In female mice with 4T1 cell xenografts, Nar treatment in combination with radiation significantly decreased tumor weight and growth rate while increasing percent survival compared to radiation alone, indicating Nar may decrease the cytotoxic effects of radiation treatment and increase efficacy [58]. Additionally, Nar treatment decreased TGF-β1_EV_ secretion from tumors treated with radiation; TGF-β1 secretion is associated with increased Treg infiltration and radiation resistance of tumors [58]. Therefore, Treg infiltration was investigated and revealed that tumors treated with Nar had decreased Treg infiltration and a higher ratio of CD8+ to Tregs, indicating a better immune response to tumors [58]. To compare Nar treatment to a positive control, the 1D11 neutralizing antibody for TGF-β1 was used; Nar-treated mice had a greater delay in tumor growth, shorter recovery time from radiation, and had greater prevention of radiation induced weight loss than 1D11 antibody-treated mice [58]. Overall, these results indicate the potential of Nar to increase radiotherapy efficacy by decreasing TGF-β1 tumor secretion, thereby decreasing intratumor Tregs and restoring the CD8+/Tregs ratio. Although an in vitro investigation into the mechanisms behind decreased TGF-β1 secretion was completed and PKC-ζ was identified, no investigation into intratumor levels of PKC-ζ was completed.

**Figure 3:**
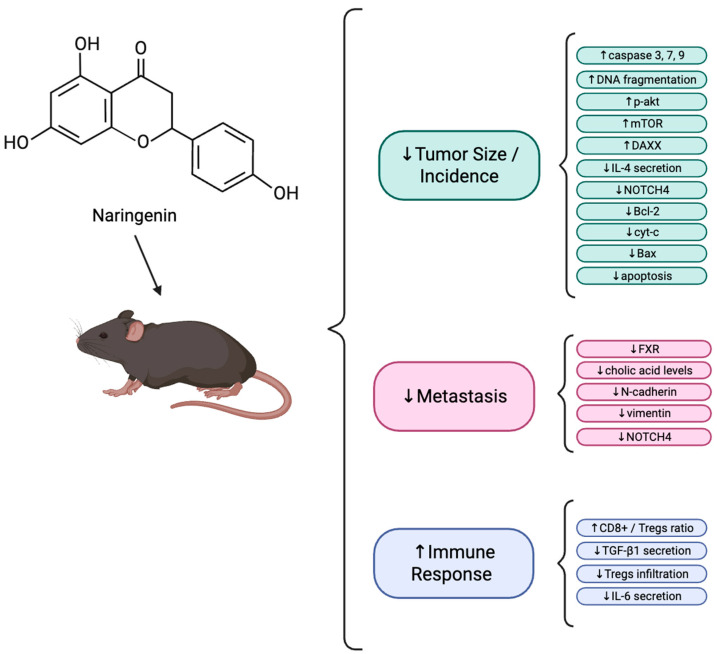
Summary of the anti-breast cancer effects of Naringenin utilizing in vivo models of breast cancer. Created in BioRender. Emily Irwin. (2026) https://BioRender.com/c29m951.

In vivo studies indicate that Nar has variable effects on breast cancer growth and metastasis depending on the experimental model, dosage, and timing of administration. In some models, Nar alone did not reduce primary tumor size, such as in 4T1 and MCF-7 xenografts, but did decrease metastasis and improve survival by enhancing CD8+ T cell activation and reducing regulatory T cells through suppression of TGF-β1 signaling [25,63,64]. In contrast, other models, including DMBA-induced breast cancer, EAC tumors, ER+ PDX models, and spontaneous mammary tumors, indicated that Nar alone decreased tumor incidence, size, and proliferation, and increased apoptosis, inhibition of colony formation, and suppression of tumor-initiating cell survival [28,29,31,66,67] (Figure 3). Combination treatments with chemotherapy, antioxidants, or radiation generally enhanced anti-tumor effects, reducing tumor volume, proliferation markers, and metastasis while minimizing toxicity [46,56,65]. These contradictory results may stem from differences in cancer models, tumor microenvironments, Nar bioavailability, dosing regimens, and timing of administration, highlighting the need for systematic studies across diverse models and clinical evaluation to clarify its therapeutic potential.

## 9. Conclusions

Over the past 15 years, a growing body of preclinical evidence from both in vitro and in vivo animal studies has highlighted the promising anti-breast cancer potential of Nar. Nar has been shown to inhibit proliferation, induce apoptosis, and suppress migration and invasion of breast cancer cells while modulating key signaling pathways such as PI3K/Akt, MAPK, and NF-κB. In various breast cancer cell lines, including ER-positive, HER2-positive, and triple-negative subtypes, Nar demonstrated the ability to regulate oxidative stress, restore apoptotic balance, interfere with growth factor signaling, and disrupt mitochondrial function (Figure 1). These effects are mediated through mechanisms including activation of caspases, regulation of pro- and anti-apoptotic proteins, induction of cell cycle arrest, and suppression of metastasis-associated factors. Emerging evidence also suggests that nanoparticle formulations and structural derivatives of Nar may enhance its anti-cancer activity, while combination approaches with conventional therapies such as chemotherapy, radiotherapy, and hormone therapy may further improve therapeutic outcomes. Animal studies further support its potential, demonstrating reduced tumor growth or metastasis and improved survival in some models with minimal toxicity to normal tissues (Figure 3). Collectively, these findings suggest that Nar exerts anti-breast cancer effects through the modulation of multiple signaling pathways involved in tumor growth, survival, and metastasis, highlighting its potential as a therapeutic agent.

Despite these promising findings, several limitations exist in the current literature. Most studies investigating Nar have been conducted in vitro using breast cancer cell lines, which cannot fully replicate the complexity of tumor biology, tumor–microenvironment interactions, and pharmacokinetics observed in vivo. Although some animal studies have examined Nar, their findings remain inconsistent, with some models demonstrating reduced tumor growth and metastasis while others report limited effects on primary tumor size. These discrepancies may result from differences in experimental models, tumor microenvironments, Nar bioavailability, dosing regimens, and timing of administration. In addition, mechanistic findings are sometimes inconsistent, particularly regarding the involvement of estrogen receptor signaling and the regulation of apoptosis-related proteins such as Bax and Bcl-2, which may vary depending on breast cancer subtype, treatment concentration, and exposure duration. Evidence supporting the use of Nar in combination with chemotherapy, radiotherapy, hormone therapy, or antioxidant compounds is also limited, often restricted to a small number of in vitro studies, with hormone therapy interactions reported in a single study. Furthermore, although nanoparticle formulations and chemical derivatives of Nar appear to enhance anti-cancer activity, their pharmacological advantages and in vivo efficacy remain insufficiently characterized. Future research should therefore focus on systematic in vivo investigations across diverse breast cancer models, clarification of the molecular mechanisms underlying Nar’s activity, and expanded evaluation of combination therapies. Ultimately, well-designed clinical studies are required to determine the safety, pharmacokinetics, and therapeutic potential of Nar in breast cancer patients.

Overall, Naringenin represents a promising plant-derived compound with multi-targeted anticancer effects. Continued investigation is warranted to fully elucidate its mechanisms and evaluate its therapeutic value in clinical settings for breast cancer management.

## Figures and Tables

**Table 2:** Evidence of the effects of Naringenin analogs and nanoparticles against breast cancer: summary of in vitro studies.

Reference	Cell Line	Treatment	Finding	Mechanism
[39]	*Saccharomyces cerevisiae* yeast	500 pM–100 µM Nar, 8PN, or 6DMAN 24 h	↑ ERα activity	N/A
	MVLN cells or Ishikawa cells	10–1000 nM Nar, 8PN, or 6DMAN 24 h or 72 h	↑ ERα activity	N/A
[40]	MDA-MB-231	1–100 µM NarCuB 24 h	↓ colony size and formation ↓ proliferation ↓ migration ↑ apoptosis	↓ Pro-MMP protein levels ↑ Apoptotic nuclei ↑ Caspase-9 mRNA
[41]	SKBr3 or MDA-MB-231	73 µM VOnar 24 h Or 20 µM VOnar 24 h	↑ apoptosis ↑ nuclear damage ↑ mitochondrial arrest ↓ BSA binding affinity	↑ Intracellular ROS ↓ MMP ↑ Caspase 3/7 activity ↑ p-H2AX protein levels ↑ LDH levels ↑ p-H3
[42]	MCF-7	50–1000 µM 24 h	↓ cell viability ↑ apoptosis	↑ Intracellular ROS ↑ DNA damage
[43]	MCF-7	*Tert*-butyl-Nar 10 or 20 µM for 24 h	↓ cell viability ↑ cell cycle arrest	↑ G1 phase ↑ G2/M phase ↓ S phase
[44]	MCF-7	Nar IC50 48 h	↓ cell viability	↑ p53 mRNA ↑ Caspase 3 mRNA ↑ Bax mRNA ↑ Bcl-2 ↑ Cyt-C mRNA
	MCF-7	4-methylpiperidine or morpholine	↓ cell viability	↑ p53 mRNA ↑ Caspase 3 mRNA ↑ Bax mRNA ↑ APAF1 mRNA
	MCF-7	Piperidine or pyrrolidine	↓ cell viability	↑ p53 mRNA ↑ Caspase 3 mRNA ↑ Bax mRNA ↑ APAF1 mRNA ↑ Cyt-C mRNA
[45]	MCF-7	NarNS	↓ cell viability	↑ intracellular ROS ↑ lipid peroxidation ↓ GSH ↓ MMP ↑ Caspase 3 activity ↑ Apoptotic morphological changes
[46]	MCF-7	CUR-NAR-D-MNPs, 59.7 μg/mL, 48 h	↓ cell viability	↑ Early and late-stage apoptosis ↑ Necrosis ↑ ROS levels
[47]	MDA-MB-231	Sub IC50 value of Nar-loaded PDNG NPs 12, 24, and 48 h	↓ cell viability	↑ Intracellular ROS ↑ Mitochondrial membrane depolarization ↑ Apoptosis ↑ G1 phase ↑ G2/M phase ↑ Caspase 3 and 9 protein levels
	MCF-7 or T47D	1, 5 and 25 μM Nar loaded PDNG NPs 48 h	↓ cell viability	N/A
[48]	MCF-7	NarSPNP (0.3 mg/mL Nar)	↓ cell viability ↑ apoptosis ↑ cell cycle arrest	↑ G1 phase ↓ S phase ↓ G2 phase
[49]	MCF-7	93.64 µM 7-methoxyl-Nar 24 h and 72 h	↓ cell proliferation ↑ cytotoxicity	N/A
[50]	MCF-7	Nar-NP 922 µg/mL 48 h	↓ cell viability	↑ subG1 phase ↑ Caspase 3, 8, and 9 mRNA levels
[51]	MDA-MB-231	CS-NPs/NAR 100, 200, or 300 µg 24 or 48 h	↓ cell viability ↓ cell proliferation	↑ Nitrate ↑ XOD ↑ ROS ↓ XDH ↑ Caspase 3 protein levels ↑ DNA fragmentation
[52]	MDA-MB-231 or MCF-7	Nar-Glucoside 233.56 µg/µL or 698.44 µg/µL 48 h	↓ cell viability	↑ DNA fragmentation ↓ EGFR cDNA levels
[53]	MDA-MB-231	Lip-Nar 546.6 µg/mL 48 h	↓ cell viability	N/A

Table legend: ↑ Increased, ↓ Decreased, p–phosphorylated.

**Table 3:** Evidence of the effects of Naringenin in Combination with chemotherapy against breast cancer: summary of in vitro studies.

Reference	Cell Line	Treatment	Finding	Mechanism
[54]	MCF-7	Nar 250 μM and/or Tam 100 nM 4–7 days 200 μM Nar and 25 nM Tam 4 days	↓ Cell viability ↓ Proliferation ↑ Apoptosis	↓ ERK1/2 and Akt protein levels ↓ Caspase 7 and 9 protein levels ↑ cleaved PARP
[55]	MDA-MB-231	Naringenin (0- 500 μM) or Cyclophosphamine (Cpm) (0–40 μM) 4–48 h ↓	↓ Cell viability ↓ Proliferation ↑ Apoptosis	↑early and late-stage apoptotic cells ↑Bax protein ↓Bcl-2 protein ↑Caspase 3 activity ↑ Caspase 9 activity ↓p-STAT3
[56]	MDA-MB-231	Nar (1.25 mM) Met (12.5 mM) DOX (1.5–120 μM)	↓ Cell viability	N/A
	4T1	Nar (0.625 mM) Met (6.25 mM) DOX (0.47–30 μM)	↓ Cell viability	N/A
[57]	HCC1806 (CRL-2335) Or 4T1	0–1024 nM PTX and Nar 48 h	↓ Cell viability	↓ IC50 value of PTX ↑ NR3C2 activation

Table legend: ↑ Increased, ↓ Decreased, p–phosphorylated.

**Table 4:** Evidence of the effects of Naringenin in combination with radiotherapy against breast cancer: summary of in vitro studies.

Reference	Cell Line	Treatment	Finding	Mechanism
[58]	4T1 breast cancer cells	Nar 200 µM, 30 min And Radiation 2, 4, or 8 Gy	↓ Treg differentiation	↓ PKC-ζ phosphorylation ↓ TGF-β1 secretion ↓ O2˙− ↓ Zinc release
[53]	MDA-MB-231	Lip-Nar 546.6 µg/mL 48 h	↓ Cell viability ↓ Colony formation	N/A

Table legend: ↓ Decreased.

**Table 5:** Evidence of the Effects of Naringenin in combination with hormone therapy against breast cancer: summary of in vitro studies.

Reference	Cell Line	Treatment	Finding	Mechanism
[59]	MCF-7	Nar (200 μM) and Tam (5 μM), 24–48 h	↓ Cell viability ↓ Migration ↓ Invasion ↑ Cell cycle arrest ↑ Apoptosis	↓ MMP-2 mRNA ↓ MMP-9 mRNA ↑ S phase ↑ G2/M phase ↑ p53 mRNA ↑ p51 mRNA ↓ cyclin E mRNA ↓ cyclin D mRNA ↑ early and late-stage apoptosis ↑ nuclear condensation ↓ Bcl-2 and Bax mRNA ↑ intracellular ROS ↓ ERα66, ERα36 and GPR30 mRNA and protein ↑ ERβ mRNA and protein

Table legend: ↑ Increased, ↓ Decreased.

**Table 6:** Evidence of the effects of Naringenin in combination with other potential natural therapeutics in breast cancer: summary of in vitro studies.

Reference	Cell Line	Treatment	Findings	Mechanism
[60]	MCF-7	Nar (0.5, 1, and 2 x IC50 value) and Balsamin (25 μg/mL) 48 h	↓ Cell viability ↑ Apoptotic morphological changes	↑ Cell shrinkage ↑ Cell surface detachment ↑ Cytoplasmic density ↑ Caspase 3 activity ↑ Caspase 9 activity ↓ Bcl-2 mRNA ↓ Bcl-XL mRNA ↑ Bax mRNA ↑ Bid mRNA ↑ Bad mRNA ↑ p53 mRNA ↑ CHOP mRNA ↑ GRP78 mRNA
[61]	MCF-7	Nar and Que 44.31 µg/mL 24 h	↓ Cell viability	↓ Bcl-2 mRNA ↓ MMP ↓ Caspase 3/7 activity ↑ Lipid peroxidation
[62]	MCF-7 or MDA-MB-231	Nar (IC10) and Que or Nar (IC30) and Fis 24–72 h	↓ Cell viability ↓ Migration	↑ Caspase 3, 8, and 9 mRNA levels ↓ Bcl-2 mRNA levels ↑ miR-1275 mRNA levels ↓ mir-27a-3p mRNA levels

Table legend: ↑ Increased, ↓ Decreased.

**Table 7:** Evidence of the effects of Naringenin against breast cancer: summary of in vivo studies.

Reference	Xenograft Model	Treatment	Findings	Mechanism
[63]	female BALB/c mice 4T1 breast cancer cells injected into the fourth mammary fat pad	Nar—100 mg/kg/day oral administration 24 days	↓ Metastasis	↑ IFN-γ and IL-2 expressing CD4+ and CD8+
[64]	Ovariectomized female athymic mice, MCF-7aro breast cancer cells injected into flanks	Nar—5000 ppm, oral administration	(No effects)	N/A
[25]	Female Balb/c mice injected with 4T1/TGF-β1 cells in the 4th mammary fat pad	200 mg/kg Naringenin once daily for 30 days	↓ Metastasis ↑ Survival	↓ TGF-β1 protein levels in tissue and serum ↓ Foxp3 protein levels ↓ CD4+ CD25+ Foxp3+ Tregs lungs ↓ CD103+ CD4+ Foxp3+ Tregs spleen ↓ CD4+ Gr11b+ T in cells lungs/spleen ↑ CD4+ CD44+ CD62L– T in cells lungs/spleen ↑ CD8+ IFNγ+ T cells in spleen ↑ IFNγ mRNA in lungs ↑ Granzyme-B mRNA in lungs
[26]	HFD-fed ovariectomized C57BL/6 mice injected with E0771 breast cancer cells in thoracic mammary fat pad	High Nar-fed 3 wt/wt % Nar 20 days	No effects on tumor size	↓ Body weight ↓ Blood glucose ↓ Calorie intake ↓ Adipose mass ↓ MCP-1 mRNA ↓ IL-6 mRNA ↑ Tumor p-Akt ↑ Tumor p-mTOR
[28]	Female Wistar rats, single intravenous injection of DMBA (8 mg/kg body weight)	Low Nar: 2.5 mg/kg body weight Medium Nar: 5 mg/kg body weight High Nar: 10 mg/kg, body weight	↓ Tumor size and weight ↓ Tumor incidence ↓ Tumor burden ↓ Body weight ↓ Lymph node and pulmonary metastases	↓ PCNA-positive cells ↑ DNA fragmentation ↑ Caspase 3/9 activity ↓ TBARS ↓ SOD levels ↓ Catalase levels ↓ Protein carbonyl levels ↓ Nitrate levels ↑ GSH levels ↑ Vitamin C levels ↑ Vitamin E levels ↑ Glutathione reductase levels ↑ Bax mRNA ↓ Bcl-xl and Bcl-2 mRNA ↓ Apaf-1 mRNA ↓ VDAC mRNA ↓ Cytochrome c mRNA ↑ Procaspase-9 mRNA
[29]	MCF-7, grown into mammosphere, injected into mammary fat pad of female, ovariectomized FoxN1 nu/nu athymic nude mice	Nar 20 mg/kg oral gavage 5 days on 2 days off 8 weeks	↓ Tumor development ↓ Tumor size	N/A
	Female ovariectomized FoxN1 nu/nu athymic mice with subcutaneous implantation into mammary fat pad of ER+ PDX tumor (BCM-5097)	Nar 20 mg/kg oral gavage 5 days on 2 days off 2 weeks	↓ Tumor size	↑ DAXX protein levels ↓ NOTCH4 protein levels ↓ MFE
[56]	Male athymic balb/c nude mice subcutaneous injection of MDA-MB-231 cells	Nar 50 mg/kg Met 100 mg/kg Oral administration Lip-Dox 3 mg/kg intravenous injection 28 days	↓ Tumor volume	↓ Serum TNF-α and IL-1β ↑ Tumor necrosis ↓ Ki-67
[46]	Female Sprague–Dawley rats single dose of 100 mg/kg body weight of DMBA in sesame oil was orally administered, post 4 weeks, 5 ppm subcutaneous injection of MPA (0.5 mg/rat) every week for 4 weeks	CUR-NAR-D-MNP 1 mg/kg via gastric intubation 3× per week 8 weeks And/or LDR (0.25 Gy)	↓ Tumor size ↑ Apoptosis ↑ Necrosis	↑ Body weight ↑ Pyknotic nuclei ↑ Large eosinophilic granular areas ↑ Apoptotic bodies ↑ Cellular debris ↑ G0/1 phase cycle arrest ↑ Bax ↑ caspase 3 ↓ Bcl-2 ↑ p53 ↑ p21 ↑ ROS ↓ TNF-α ↓ CD44
[31]	Immature female Swiss albino mice	Nar 30 mg/kg, i.p and/or Estradiol 12.5 µg/kg oral 7 days daily	Delayed vaginal opening Delayed vaginal cornification ↓ Uterine weight	N/A
	Mature female Swiss albino mice with Ehrlich ascites carcinoma (EAC), transplanted to hind limb	Nar 50 mg/kg, i.p, daily 20 days	↓ Tumor size	↓ Aromatase activity
[47]	Sparge–Dawley rats	PDNG NPs (1 mg/kg body weight) iv bolus dose 12 h	↑ Bioavailability ↑ Half-life	N/A
	Female nude mice, fed with water containing estradiol (0.3 mg/mL) for 1 week pre subcutaneous injection of MCF-7 breast cancer cells in right forelimb	Nar 1 mg/kg every 3 days iv bolus dose 40 days	↓ Tumor size ↓ Body weight	N/A
[65]	Female Sprague–Dawley rats MNU-induced breast carcinoma (50 mg/kg MNU intraperitoneally)	Nar (50 mg/kg) and metformin (100 mg/kg) orally administered 28 days and intravenous injection of liposomal doxorubicin (2 mg/kg) every 7 days	↓ Tumor incidence ↓ Tumor number ↓ Tumor multiplicity ↓ Tumor weight ↑ Tumor necrotic area	N/A
	4T1 breast cancer cells xenograft in female Balb/c mice	Nar (50 mg/kg) and metformin (100 mg/kg) orally administered 28 days and intravenous injection of liposomal doxorubicin (3 mg/kg) every 7 days	↓ Tumor weight ↑ Antitumor activity ↓ Toxicity ↑ Tumor necrotic area	N/A
[33]	Male BALB/c nude mice, T47D subcutaneous injection into flank	Nar 50 mg/kg administered orally 28 days	↓ Tumor weight ↓ Tumor size	N/A
[66]	Female Balb/c mice with explants of spontaneous mouse mammary tumor (SMMT) subcutaneously implanted	Nar 50 mg/kg/day and CPT 20 mg/kg/day Intraperitoneal (IP) injection	↓ Tumor size	↑ Splenocyte proliferation ↓ Splenocyte IL-4 secretion ↑ Splenocyte IFN-γ secretion ↓ Splenic and tumor CD4+ CD25+ Foxp3+ T cells ↓ JAK/STAT phosphorylation ↓ Bcl-XL and Mcl-1 mRNA
[67]	Female Sprague–Dawley rats with DMBA-induced breast cancer	Naringenin (3.5 mg/mL) administered to the animals with an intragastric tube at a volume of 0.4 mL. (90 days)	↓ Tumor incidence ↓ Tumor number Delayed tumor development	N/A
[38]	Zebrafish breast cancer xenotransplantation model with E0771 cell injection	Cortisol 200 nM Nar 10–100 nM	↓ Tumor size ↓ Metastasis	↓ Estradiol ↑ E2-3-O-sulfate ↓ FXR protein expression ↑ EST protein expression
	Female C57BL/6 mice with orthotopic breast cancer model by injection of E0771 breast cancer cells into 4th mammary fat pads under CUMS	Nar 20 mg/kg by oral gavage	↓ Depression-like symptoms ↓ Tumor size ↓ Metastasis	↑ Mobility time ↑ Sucrose preference ↓ N-cadherin protein levels ↓ Vimentin protein levels ↓ Ki67 protein levels ↓ Estradiol ↑ E2-3-O-sulfate ↓ FXR protein expression ↑ EST protein expression
[58]	Female Balb/c mice subcutaneous injection of 4T1	Radiotherapy: 10 Gy Nar 100 mg/kg/day intragastric (30 days)	↓ Tumor size ↓ Tumor growth rate ↑ % survival	↓ TGF-β1_EV_ secretion ↓ Tregs infiltration ↑ CD8+/Tregs ratio

Table legend: ↑ Increased, ↓ Decreased, p–phosphorylated.

## Data Availability

No new data were created or analyzed in this study.
